# Clinical presentation of young people (10–24 years old) with brain tumors: results from the international MOBI-Kids study

**DOI:** 10.1007/s11060-020-03437-4

**Published:** 2020-03-03

**Authors:** Angela Zumel-Marne, Michael Kundi, Gemma Castaño-Vinyals, Juan Alguacil, Eleni Th Petridou, Marios K. Georgakis, Maria Morales-Suárez-Varela, Siegal Sadetzki, Sara Piro, Rajini Nagrani, Graziella Filippini, Hans-Peter Hutter, Rajesh Dikshit, Adelheid Woehrer, Milena Maule, Tobias Weinmann, Daniel Krewski, Andrea ′t Mannetje, Franco Momoli, Brigitte Lacour, Stefano Mattioli, John J. Spinelli, Paul Ritvo, Thomas Remen, Noriko Kojimahara, Amanda Eng, Angela Thurston, Hyungryul Lim, Mina Ha, Naohito Yamaguchi, Charmaine Mohipp, Evdoxia Bouka, Chelsea Eastman, Roel Vermeulen, Hans Kromhout, Elisabeth Cardis

**Affiliations:** 1 ISGlobal, Barcelona, Spain; 2 Universitat Pompeu Fabra (UPF), Barcelona, Spain; 3 Ciber Epidemiología y Salud Pública (CIBERESP), Madrid, Spain; 4 Center for Public Health, Department of Environmental Health, Medical University Vienna, Vienna, Austria; 5 IMIM (Hospital del Mar Medical Research Institute), Barcelona, Spain; 6 Environmental Epidemiology and Neuroscience Laboratory, RENSMA, Huelva University, Av. Andalucía s/n, E-21071 Huelva, Spain; 7 Department of Hygiene, Epidemiology and Medical Statistics, Medical School, National and Kapodistrian University of Athens, Athens, Greece; 8 Clinical Epidemiology Unit Karolinska Institutet, Stockholm, Sweden; 9 Área de Medicina Preventiva y Salud Pública, Universitat de Valencia, Valencia, Spain; 10 Public Health Services, Ministry of Health, Tel Aviv, Israel; 11 Sackler Faculty of Medicine, Tel Aviv University, Tel Aviv, Israel; 12 Environmental and Occupational Epidemiology Branch, Cancer Risk Factors and Lifestyle Epidemiology Unit, Institute for Cancer Research Prevention and Clinical Network-ISPRO, Florence, Italy; 13 Centre for Cancer Epidemiology, Tata Memorial Centre, Mumbai, India; 14 Leibniz Institute for Prevention Research and Epidemiology - BIPS, Bremen, Germany; 15 Scientific Director’s Office, Fondazione IRCCS Istituto Neurologico Carlo Besta, Milan, Italy; 16 Institute of Neurology, Medical University Vienna, Vienna, Austria; 17 Unit of Cancer Epidemiology, Department of Medical Sciences, University of Turin, Turin, Italy; 18 Institute and Clinic for Occupational, Social and Environmental Medicine, University Hospital, LMU Munich, Munich, Germany; 19 School of Epidemiology and Public Health, University of Ottawa, Ottawa, Canada; 20 Centre for Public Health Research, Massey University, Wellington, New Zealand; 21 French National Registry of Childhood Solid Tumors, CHU, Nancy, France; 22 Inserm, Center of Research in Epidemiology and StatisticS (CRESS), Epidemiology of Childhood and Adolescent Cancers Team (EPICEA), Paris University, Paris, France; 23 Department of Medical and Surgical Sciences (DIMEC), University of Bologna, Bologna, Italy; 24 Population Oncology, BC Cancer, Vancouver, Canada; 25 School of Population and Public Health, University of British Columbia, Vancouver, Canada; 26 School of Kinesiology and Health Science and Department of Psychology, York University, Toronto, Canada; 27 Department of Public Health, Tokyo Women’s Medical University, Tokyo, Japan; 28 Department of Preventive Medicine, Dankook University College of Medicine, Cheonan, South Korea; 29 Children’s Hospital of Eastern Ontario, Ottawa, ON Canada; 30 University of Ottawa, Ottawa, ON Canada; 31 Division Environmental Epidemiology, Institute for Risk Assessment Sciences, Utrecht University, Utrecht, The Netherlands

**Keywords:** Brain tumor, Diagnosis, Symptom, Central nervous system tumor, Clinical characteristic

## Abstract

**Introduction:**

We used data from MOBI-Kids, a 14-country international collaborative case–control study of brain tumors (BTs), to study clinical characteristics of the tumors in older children (10 years or older), adolescents and young adults (up to the age of 24).

**Methods:**

Information from clinical records was obtained for 899 BT cases, including signs and symptoms, symptom onset, diagnosis date, tumor type and location.

**Results:**

Overall, 64% of all tumors were low-grade, 76% were neuroepithelial tumors and 62% gliomas. There were more males than females among neuroepithelial and embryonal tumor cases, but more females with meningeal tumors. The most frequent locations were cerebellum (22%) and frontal (16%) lobe. The most frequent symptom was headaches (60%), overall, as well as for gliomas, embryonal and ‘non-neuroepithelial’ tumors; it was convulsions/seizures for neuroepithelial tumors other than glioma, and visual signs and symptoms for meningiomas. A cluster analysis showed that headaches and nausea/vomiting was the only combination of symptoms that exceeded a cutoff of 50%, with a joint occurrence of 67%. Overall, the median time from first symptom to diagnosis was 1.42 months (IQR 0.53–4.80); it exceeded 1 year in 12% of cases, though no particular symptom was associated with exceptionally long or short delays.

**Conclusions:**

This is the largest clinical epidemiology study of BT in young people conducted so far. Many signs and symptoms were identified, dominated by headaches and nausea/vomiting. Diagnosis was generally rapid but in 12% diagnostic delay exceeded 1 year with none of the symptoms been associated with a distinctly long time until diagnosis.

**Electronic supplementary material:**

The online version of this article (10.1007/s11060-020-03437-4) contains supplementary material, which is available to authorized users.

## Introduction

Brain tumors (BT) are one of the most common tumor types in young people. In 2018, the estimated age-standardized annual rate for brain and central nervous system (CNS) tumors in high income areas was 2.5 per 100,000 persons below the age of 25 [1]. Incidence of BT has risen during the last decades and it is unclear whether this is due solely to improved diagnostic practices or to a real increase in disease frequency [2, 3].

Tumor histology and location vary with age. In adults, the most frequent BT subtypes are gliomas located in the supratentorial region—and specifically in the temporal and parietal lobes—while in children, the most frequent are low grade gliomas [4] and embryonal tumors located primarily in the posterior fossa (cerebellum and brainstem) [5].

Symptoms preceding BT diagnosis are non-specific, and are often initially attributed to other diseases. Advanced knowledge about common symptoms of BT in the pediatric, adolescent and young adult populations could raise awareness among the medical community [6, 7].

The primary objective of this paper is to describe the clinical characteristics of BT in young people using data from 899 cases recruited in the international MOBI-Kids case–control study, focusing on morphology, topography, signs and symptoms and time to diagnosis. We also aim to study whether certain signs and symptoms are associated with specific tumor morphologies and locations, or with variables such as gender or age. We report results for older children (10 years or older), adolescents and young adults (up to the age of 24) (a group referred to as “young people” in this paper) to provide information for those studying childhood as well as young adult oncology.

## Material and methods

MOBI-Kids is a multinational case–control study set-up to estimate risk of BTs in relation to electromagnetic fields—mainly Extremely Low Frequency and radiofrequency (RF) fields—exposure from use of mobile communication device. Cases were patients with a first primary benign or malignant BT, diagnosed between the ages of 10 and 24 years, during a 3–4 study period between 2010–2016 (depending on country), and residing in one of the study regions of the participating countries (Australia, Austria, Canada, France, Germany, Greece, India, Israel, Italy, New Zealand, Spain, Netherlands, Japan and Korea). Eligible tumors were those originating in areas of the brain that absorb the highest RF energy from mobile phones held by the ear, thus excluding midline tumors (those close to the skull base, mostly pituitary and pineal tumors). Tumors known to be associated with a genetic syndrome were also excluded. Ethics committee approvals for the study were obtained from all national and regional review boards. An informed consent was obtained from all participants. The detailed protocol of MOBI-Kids was previously published [8].

A clinical questionnaire was completed by interviewers trained for this study with the help of neurosurgeons, neuroradiologists and/or pathologists, based on available clinical records including imaging, histopathology, surgery, and on clinical reports. Quality and precision of reports about clinical presentation especially concerning timing of signs and symptoms varied but were typically more precise the closer to diagnosis they occurred. There were some differences among countries concerning participation of neurosurgeons or pathologists in data extraction. Examination of precision and completeness revealed no systematic differences with respect to their participation. In cases where ambiguities could not be solved, the team reviewed the clinical records a second time. In this article we explore the information from the clinical questionnaire concerning the clinical characteristics of the tumors in MOBI-Kids.

Details about data collected, coding and statistical methods are provided in the Supplemental Materials.

## Results

**Fig. 1:**
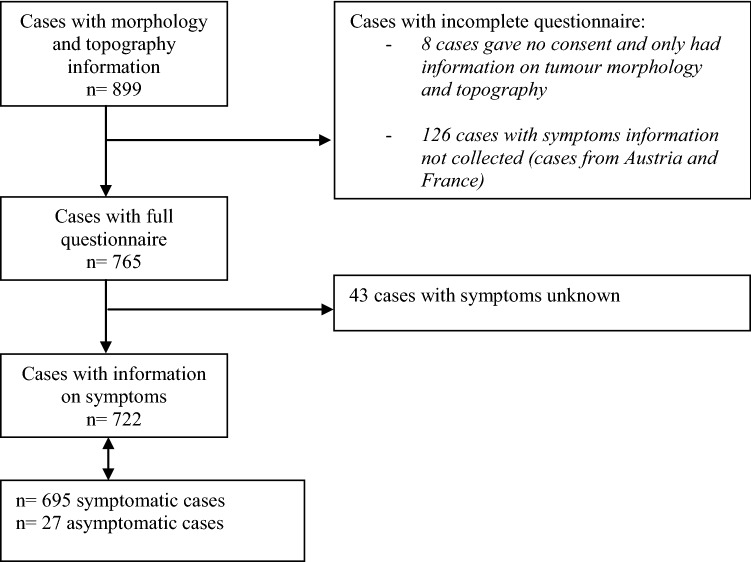
Flowchart for collection of information

We collected clinical information on 899 cases. Eight did not authorize access to their clinical records and hence the only information available is tumor morphology and topography. Symptoms information was available for 722 cases (two participating countries did not collect information about these) (Fig. 1). Overall, 42% of cases were aged 10–14 years at diagnosis, 32% 15–19 years and 26% 20–24 years (Table 1), with a higher proportion of males (57%) than females.

**Table 1:** Number of cases and cases with reported symptoms, median number of symptoms by age, sex, morphology, topography and grade of tumor

	Cases (n = 899)^a^	Cases with symptom information (n = 722)^b^	Symptomatic cases (n = 695)^c^	Number of symptoms reported (n = 695)	
	n (%)	n (%)	n (%)	Median (IQR)	p-value^d^
Age					< 0.01
10–14	375 (42)	301 (42)	292 (97)	3 (2–4)	
15–19	292 (32)	230 (32)	221 (96)	2 (2–3)	
20–24	232 (26)	191 (26)	182 (95)	2 (1–3)	
Sex					0.03
Male	512 (57)	397 (55)	377 (95)	2 (2–4)	
Female	387 (43)	325 (45)	318 (98)	3 (2–4)	
Morphology					< 0.01
Gliomas	556 (62)	459 (64)	443 (97)	2 (2–4)	
Other neuroepithelial	120 (14)	92 (13)	90 (98)	2 (1–3)	
Embryonal	129 (14)	102 (14)	98 (96)	4 (2–5)	
Meningiomas	47 (5)	35 (5)	32 (91)	2 (1–3)	
Other non-neuroepithelial	47 (5)	34 (4)	32 (94)	3 (2–4)	
Topography					< 0.01
Brain stem	91 (10)	68 (9)	65 (96)	3 (2–5)	
Cerebellum	201 (22)	163 (23)	157 (96)	3 (2–4)	
Frontal lobe	143 (16)	113 (16)	106 (94)	2 (1–3)	
Temporal lobe	103 (11)	88 (12)	87 (99)	2 (1–3)	
Parietal lobe	52 (6)	37 (5)	36 (97)	2.5 (2–3)	
Occipital lobe	17 (2)	16 (2)	15 (94)	2 (1–3)	
Cerebral ventricles	88 (10)	74 (10)	73 (99)	3 (2–4)	
Cerebral meninges	31 (4)	24 (3)	23 (96)	2 (1–3)	
Cranial nerves	30 (3)	21 (3)	20 (95)	2 (1–4)	
Overlapping lesion of the brain	83 (9)	76 (11)	73 (96)	3 (1–5)	
Other parts of the brain^d^	60 (7)	42 (6)	40 (95)	2 (2–3)	
Grade of tumor (WHO)					< 0.01
I	386 (43)	308 (42)	295 (96)	2 (1–4)	
II	177 (20)	144 (20)	137 (95)	2 (1–3)	
III	142 (15)	113 (16)	111 (98)	3 (2–4)	
IV	194 (22)	157 (22)	152 (97)	3 (2–4)	
Overall	899	722	695	3 (2–4)	

^a^Percentage calculated based on the total number of 899 cases; ^b^percentage calculated based on the total number of 722 cases with information on symptoms; ^c^percentage calculated based on row category number of cases with symptom information; ^d^Kruskal–Wallis test comparing number of reported symptoms; ^d^including brain NOS; *IQR* inter quartile range

### Tumor characteristics

The majority of cases had neuroepithelial tumors (76%) (Table 1). Gliomas represented 62% of all tumors; other neuroepithelial tumors were a variety of different morphologies, such as ganglioglioma NOS (n = 55), dysembryoplastic neuroepithelial tumors (n = 23), central neurocytomas (n = 18), among other rare tumors. Among non-neuroepithelial tumors, embryonal tumors were the most frequent (14% of all tumors), followed by meningioma (5%). Cases with rare morphologies (≤ 30 cases) are described in Supplementary Tables A13–A14.

The distribution of neuroepithelial tumors was similar across age-groups and sex overall (Table 2). Embryonal tumors were more frequent among males and a significant decreasing trend was seen with increasing age. In contrast, meningiomas and other non-neuroepithelial tumors were slightly more frequent among females, with frequency increasing with age.

**Table 2:** Morphology and topography by age and sex

	Overall				10–14 years old				15–19 years old				20–24 years old				p-value^b^
	Total [n = 899 (%)]	Male [n = 512 (%)]	Female [n = 387 (%)]	p-value^a^	Total [n = 375 (%)]	Males [n = 213 (%)]	Females [n = 162 (%)]	p-value^a^	Total [n = 292 (%)]	Males [n = 156 (%)]	Females [n = 136 (%)]	p-value^a^	Total [n = 232 (%)]	Males [n = 143 (%)]	Females [n = 89 (%)]	p-value^a^	
Morphology																	
Neuroepithelial																	
All	676 (76)	373 (73)	303 (78)		288 (77)	154 (72)	134 (83)		219 (75)	113 (72)	106 (78)		169 (73)	106 (74)	63 (71)		
Gliomas	556 (62)	304 (59)	252 (65)	0.57	234 (62)	119 (56)	115 (71)	0.06	173 (59)	88 (56)	85 (63)	0.68	149 (64)	97 (68)	52 (58)	0.08	0.11
Gliomas-high grade^d^	201 (22)	111 (22)	90 (23)	0.85	73 (19)	41 (19)	32 (20)	0.27	61 (21)	27 (17)	34 (25)	0.20	67 (29)	43 (30)	24 (27)	0.83	0.02
Gliomas-low grade^d^	355 (40)	193 (38)	162 (42)		161 (43)	78 (37)	83 (51)		112 (38)	61 (39)	51 (38)		82 (35)	54 (38)	28 (32)		
Other neuroep	120 (14)	69 (14)	51 (13)		54 (15)	35 (16)	19 (12)		46 (16)	25 (16)	21 (15)		20 (9)	9 (6)	11 (12)		
Non-neuroepithelial																	
All	223 (24)	139 (27)	84 (22)	< 0.01	87 (23)	59 (28)	28 (17)	0.44	73 (25)	43 (28)	30 (22)	0.03	63 (27)	37 (26)	26 (29)	0.15	
Embryonal	129 (14)	90 (18)	39 (10)		72 (19)	48 (23)	24 (15)		40 (14)	29 (19)	11 (8)		17 (7)	13 (9)	4 (4)		< 0.01
Meningiomas	47 (5)	21 (4)	26 (7)		4 (1)	2 (1)	2 (1)		21 (7)	9 (6)	12 (9)		22 (9)	10 (7)	12 (13)		< 0.01
Other non-neuroepithelial	47 (5)	28 (5)	19 (5)		11 (3)	9 (4)	2 (1)		12 (4)	5 (3)	7 (5)		24 (10)	14 (10)	10 (11)		< 0.01
Topography																	
Brain stem	91 (10)	52 (10)	39 (10)	0.35	54 (14)	33 (15)	21 (13)	0.25	25 (9)	11 (7)	14 (10)	0.35	12 (5)	8 (6)	4 (4)	0.89	< 0.01
Cerebellum	201 (22)	112 (22)	89 (23)		102 (27)	52 (24)	50 (31)		62 (21)	39 (25)	23 (17)		37 (16)	21 (15)	16 (18)		< 0.01
Frontal lobe	143 (16)	94 (18)	49 (13)		37 (10)	21 (10)	16 (10)		50 (17)	33 (21)	17 (13)		56 (24)	40 (28)	16 (18)		< 0.01
Temporal lobe	103 (11)	55 (11)	48 (12)		*32 (9)*	17 (8)	15 (9)		37 (13)	17 (11)	20 (15)		34 (15)	21 (15)	13 (15)		0.02
Parietal lobe	52 (6)	31 (6)	21 (5)		*23 (6)*	15 (7)	8 (5)		18 (6)	9 (6)	9 (7)		11 (5)	7 (5)	4 (4)		0.51
Occipital lobe	17 (2)	8 (2)	9 (2)		*6 (2)*	2 (1)	4 (2)		8 (3)	5 (3)	3 (2)		3 (1)	1 (1)	2 (2)		0.93
Cerebral ventricles	88 (10)	51 (10)	37 (10)		*37 (10)*	27 (13)	10 (6)		33 (11)	14 (9)	19 (14)		18 (8)	10 (7)	8 (9)		0.49
Cerebral meninges	31 (4)	14 (3)	17 (4)		*2 (0)*	1 (0)	1 (1)		12 (4)	4 (3)	8 (6)		17 (7)	9 (6)	8 (9)		< 0.01
Cranial nerves	30 (3)	17 (3)	13 (3		*10 (3)*	7 (3)	3 (2)		6 (2)	2 (1)	4 (3)		14 (6)	8 (6)	6 (7)		0.04
Overlapping lesion of the brain	83 (9)	41 (8)	42 (11)		*47 (12)*	22 (10)	25 (15)		15 (5)	7 (4)	8 (6)		21 (9)	12 (8)	9 (10)		0.07
Other parts of brain^c^	60 (7)	37 (7)	23 (6)		*25 (7)*	16 (8)	9 (6)		26 (9)	15 (10)	11 (8)		9 (4)	6 (4)	3 (3)		0.29

^a^Comparison of males and females by chi^2^; ^b^trend test of each morphology or topography by age groups; ^c^including brain NOS; percentage by column; ^d^High grade (grade I–II) and Low grade (grade III–IV)

Regarding topography (Table 2 and Supplementary Figure A1), the most frequent locations were cerebellum, frontal and temporal lobes. The distribution of cases by gender was generally similar by topography. While the proportions of brainstem and cerebellar tumors declined with increasing age, those of tumors of the frontal lobe and cerebral meninges tumors increased with age (Table 2). Gliomas were most frequently located in the frontal lobe (20%) and cerebellum (17%), while other neuroepithelial tumors arose mainly in the temporal (32%) and frontal (16%) lobes and cerebral ventricles (17%) (Supplementary Table A3). Embryonal tumors occurred mainly in the cerebellum (67%), while the majority of other non-neuroepithelial tumors, except meningioma, were located in cranial nerves (36%).

The majority of tumors were low-grade (WHO grades I/II): 69% of neuroepithelial tumors (gliomas: 64%, other neuroepithelial: 96%), 98% of meningiomas and 100% of other non-neuroepithelial tumors. All embryonal tumors were high-grade (Supplementary Table A4). For cerebellum, brainstem, and cerebral meninges the frequency of low- and high-grade tumors were similar.

### Analysis of symptoms

Among those with information on symptoms (722 cases), 27 cases were asymptomatic, 165 cases reported 1 symptom, 177 cases 2, 153 cases 3, and 200 more than 3 symptoms (up to 10) before diagnosis. Overall, among symptomatic cases, a median of 3 symptoms was observed (Table 1); the median was lower (2) in cases 15–24 years old, than in younger cases (p < 0.01) (Table 1). Females reported more symptoms (median = 3) than males (median = 2).

Statistically significant differences in number of symptoms by morphology were observed. Cases with embryonal tumors had more symptoms (median = 4), than cases with other morphologies (median = 2–3). Tumors in the frontal, temporal, occipital lobes, cerebral meninges, cranial nerves and brain NOS had fewer symptoms (median = 2) than those located in the brainstem, cerebellum, cerebral ventricles and overlapping lesion of the brain (median = 3), or parietal lobe (median = 2.5). The number of symptoms tended to be higher in higher grade tumors (Table 1).

For those with at least one symptom, the most frequently reported were headaches (n = 436; 60% of cases with symptom information), focal neurological signs and symptoms (n = 287; 40%), nausea/vomiting (n = 277; 38%) and visual signs and symptoms (n = 217; 30%) (Table 3).

**Table 3:** Symptoms by topography

Symptoms	Topography												
	Total cases with symptom information (n = 722)	Brain stem (n = 68)	Cerebellum (n = 163)	Frontal lobe (n = 113)	Temporal lobe (n = 88)	Parietal lobe (n = 37)	Occipital lobe (n = 16)	Cerebral ventricles (n = 74)	Cerebral meninges (n = 24)	Cranial nerves (n = 21)	Overlapping lesion of brain (n = 76)	Other parts of brain^a^ (n = 42)	p-value comparing topographies
	n (%)	n (%)	n (%)	n (%)	n (%)	n (%)	n (%)	n (%)	n (%)	n (%)	n (%)	n (%)	
Headaches	436 (60)	45 (66)	134 (82)	48 (42)	36 (41)	18 (49)	7 (44)	57 (77)	10 (42)	9 (43)	44 (58)	28 (67)	< 0.01
Nausea/vomiting	277 (38)	32 (47)	103 (63)	33 (29)	15 (17)	11 (30)	3 (19)	35 (47)	1 (4)	2 (10)	28 (37)	14 (33)	< 0.01
Visual signs and symptoms	217 (30)	19 (28)	48 (29)	13 (12)	12 (14)	8 (22)	6 (38)	37 (50)	16 (67)	9 (43)	30 (39)	19 (45)	< 0.01
Focal neurological signs and symptoms	287 (40)	44 (65)	77 (47)	36 (32)	27 (31)	9 (24)	3 (19)	24 (32)	8 (33)	8 (38)	39 (51)	12 (29)	< 0.01
Cognitive, memory and behavioral change	88 (12)	9 (13)	17 (10)	9 (8)	14 (16)	5 (14)	4 (25)	9 (12)	1 (4)	2 (10)	12 (16)	6 (14)	0.56
Convulsions/seizures	174 (24)	2 (3)	6 (4)	57 (50)	54 (61)	16 (43)	7 (44)	8 (11)	6 (25)	1 (5)	15 (20)	2 (5)	< 0.01
Altered consciousness	101 (14)	5 (7)	14 (9)	22 (19)	23 (26)	6 (16)	2 (13)	9 (12)	2 (8)	1 (5)	12 (16)	5 (12)	0.01
Dizziness	141 (20)	23 (34)	48 (29)	13 (12)	10 (11)	9 (24)	2 (13)	16 (22)	2 (8)	3 (14)	10 (13)	5 (12)	< 0.01
Altered sensibility	67 (9)	9 (13)	8 (5)	8 (7)	4 (5)	4 (11)	0 (0)	13 (18)	2 (8)	7 (33)	7 (9)	5 (12)	< 0.01
Asymptomatic	27 (4)	3 (4)	6 (4)	7 (6)	1 (1)	1 (3)	1 (6)	1 (1)	1 (4)	1 (5)	3 (4)	2 (5)	0.86

Percentages by column; cases can have more than one symptom; ^a^including brain NOS

Distributions of cases by detailed symptoms and morphology are in Supplementary Table A5. The majority of glioma cases reported headaches (64% in high-grade, 59% in low-grade tumors), whereas the most frequent symptoms reported for other neuroepithelial tumors were convulsions/seizures (53%) and headaches (46%). Among non-neuroepithelial tumors, headaches were also the most frequent, except for meningiomas, with visual signs and symptoms (49%).

Distribution of cases by symptoms and topography are shown in Table 3 and, in details, in Supplementary Table A6 and Figure A2. The frequency of cognitive, memory and behavioral changes did not differ by topography. Headache was reported in 58–82% of tumors of the brainstem, cerebellum, cerebral ventricles, overlapping lesion of the brain and for brain NOS; the proportion was lower in other anatomical regions of the brain. Nausea and vomiting was reported by 63% of patients with a tumor in the cerebellum, a higher proportion than in other locations. Focal neurological signs and symptoms were mentioned for 65% of patients with a tumor in the brainstem. Convulsions/seizures were reported for 43–61% of tumors in the frontal, temporal, parietal and occipital lobes but in 5% or less of tumors of the brainstem, cerebellum, cranial nerves and brain NOS. Dizziness affected mainly cases with brainstem (34%) or cerebellar (29%) tumors.

Distributions of symptoms by grade are shown in Table 4. The proportion of cases with headaches, nausea and vomiting and dizziness varied by grade, being highest for grade IV tumors (headaches: 75%; nausea and vomiting: 61%; dizziness: 27%). In contrast, the proportion of cases with convulsions/seizures was lowest in high-grade tumors (12%). Other symptoms showed little differences by grade. Most asymptomatic tumors (48%) were grade I and of small size (mean = 2.50 cm).

**Table 4:** Symptoms by grade of tumor

Symptoms	Grade of tumor (WHO)					p-value^a^
	Total cases with symptom information (n = 722)	I [n = 308 (%)]	II [n = 144 (%)]	III [n = 113 (%)]	IV [n = 157 (%)]	
Headaches	436	181 (59)	69 (48)	68 (60)	118 (75)	< 0.01
Nausea/vomiting	277	101 (33)	34 (24)	47 (42)	95 (61)	0.11
Visual signs and symptoms	217	93 (30)	33 (23)	37 (33)	54 (34)	0.11
Focal neurological signs and symptoms	288	115 (37)	50 (35)	53 (47)	70 (45)	0.07
Cognitive, memory and behavioral change	88	37 (12)	13 (9)	16 (14)	22 (14)	0.39
Convulsions/seizures	174	75 (24)	58 (41)	22 (20)	19 (12)	< 0.01
Altered consciousness	101	48 (16)	28 (19)	8 (7)	17 (11)	0.02
Dizziness	141	54 (18)	16 (11)	28 (25)	43 (29)	< 0.01
Altered sensibility	67	27 (9)	16 (11)	15 (13)	9 (6)	0.13
Asymptomatic	27	13 (4)	*7 (5)*	2 (2)	5 (3)	0.63

Percentages by row; ^a^p-value for comparison of grades; cases can have more than one symptom

Laterality of symptoms was reported for only 17% of cases with lateralized symptoms (Supplementary Table A7). Little difference was seen for these symptoms, based on small numbers of cases, except for altered sensitivity, more frequent on the side contralateral to the tumor.

In cluster analyses of the main symptom categories (Supplementary Figures A3–A5), headaches and nausea/vomiting were the only symptoms which met the 50% statistical cut-off, occurring together for the majority of topographies and morphologies (Dice dissimilarity coefficient, DDC = 0.33, equivalent to 67% joint occurrences). Other, non- significant observations include headaches with visual signs and symptoms and focal signs and symptoms in embryonal tumors; headaches with visual signs and symptoms and nauseas/vomiting with dizziness in meningiomas; cognitive changes with convulsions/seizures in 'other non-neuroepithelial tumors’; headaches with visual signs and symptoms, dizziness and focal signs and symptoms and convulsions/seizures with altered consciousness in brainstem tumors; headaches, nausea/vomiting and focal signs and symptoms in cerebellar tumors; cognitive changes and convulsions/seizures in occipital lobe tumors.

### Time between symptoms onset and diagnosis

Time between occurrence of symptoms and final diagnosis was available for 91% of the cases (Table 5). Most (52%) were diagnosed within 2 months of first reported symptom, while 12% were diagnosed over 1 year later; of these, half were diagnosed over 2 years after first symptoms, including 13 cases 5 years or more later.

**Table 5:** Time between symptom occurrence and final diagnosis

Symptoms	Total symptomatic cases (n = 695)	Time between first symptom and final diagnosis								NA^c^
		n^a^	Months median (IQ^b^ range)	0–1 months [n (%)]	1–2 months [n (%)]	2–6 months [n (%)]	6 months to < 1 year [n (%)]	1 to 2 years [n (%)]	> 2 years^d^ [n (%)]	
Headaches	436	413	1.23 (0.50–3.33)	181 (42)	78 (18)	83 (19)	26 (6)	23 (5)	22 (5)	23 (5)
Nausea/vomiting	277	267	0.97 (0.33–2.60)	135 (49)	47 (17)	48 (17)	19 (7)	10 (4)	8 (3)	10 (4)
Visual signs and symptoms	217	148	0.62 (0.17–1.55)	93 (43)	21 (10)	16 (7)	8 (4)	7 (3)	3 (1)	69 (32)
Focal neurological signs and symptoms	288	251	0.73 (0.23–2.73)	140 (49)	35 (12)	41 (14)	15 (5)	10 (3)	10 (3)	36 (13)
Cognitive, memory and behavioral change	88	64	0.78 (0.37–3.33)	34 (39)	6 (7)	13 (15)	7 (8)	2 (2)	2 (2)	24 (27)
Convulsions/seizures	175	162	1.03 (0.27–4.27)	80 (46)	19 (11)	29 (17)	18 (10)	6 (3)	10 (6)	12 (7)
Altered consciousness	101	99	0.63 (0.17–3.33)	59 (58)	10 (10)	16 (16)	7 (7)	2 (2)	5 (5)	2 (2)
Dizziness	141	116	1.33 (0.43–3.40)	50 (35)	23 (16)	25 (18)	8 (6)	7 (5)	3 (2)	25 (18)
Altered sensibility	67	47	1.30 (0.17–5.07)	21 (31)	3 (4)	14 (21)	2 (3)	5 (7)	2 (3)	20 (30)
Overall	695	638	1.42 (0.53–4.80)	253 (36)	113 (16)	137 (20)	55 (8)	39 (6)	41 (6)	57 (8)

^a^Number with information about time before diagnosis; percentage by row; cases can have more than one symptom; ^b^IQ = interquartile; ^c^NA = date of symptom occurrence not available; ^d^The latter group had no specific characteristic: median age 17 years old; 6 gliomas, 3 other neuroepithelial, 2 embryonal tumors and 2 meningiomas; most frequent symptoms: headaches (n = 7), convulsions/seizures (n = 8), nausea/vomiting (n = 4) and visual signs and symptoms (n = 4)

The median time between the earliest symptom and diagnosis ranged from 0.80 (for visual signs and symptoms and altered consciousness) to 2.7 months (for altered sensitivity) (Supplementary Table A10). Among neuroepithelial tumors, gliomas had a shorter median time (1.10 months and 1.37 months for high- and low-grade tumors respectively) than other tumors (2.57 months). We did not find statistical differences among non-neuroepithelial tumors (Supplementary Table A8).

Regarding topography, the median time ranged from 1.07 to 1.83 months for brainstem, cerebellar, frontal lobe, cerebral ventricles, cerebral meninges, brain NOS and occipital lobe; it was somewhat longer for other tumors: 2–2.2 months for tumors in the temporal and parietal lobes, 3 months for cranial nerves tumors (Supplementary Table A8).

A common sequence of symptoms was observed in most morphologies and topographies: if headache was the first symptom, nausea/vomiting was the most frequent concurrent or following symptom, and focal neurological signs and symptoms the third (Supplementary Table A12).

We also analyzed time from the earliest and latest symptoms to diagnosis (Supplementary Table A10). The earliest symptom was headaches in 59% of cases overall (median time to diagnosis of 1.27 months), 52% of gliomas and 73% of embryonal tumors. Other frequent earliest symptoms were focal and neurological signs and symptoms (27%) and nausea/vomiting (33%). Median time was shortest for visual signs and symptoms and altered consciousness (0.8 months). The longest median time was observed for most morphologies when altered sensitivity was the earliest symptom. For embryonal tumors it was for convulsions/seizures and for meningioma cognitive, memory and behavioral changes. For the latest symptom (Supplementary Table A10), headache was the most frequently reported (47%) for all tumors except meningioma and other neuroepithelial tumors. Other frequently registered latest symptoms were nausea/vomiting (33%) and focal neurological signs and symptoms (32%). Median time to diagnosis was shortest for cases whose latest symptom was visual sign and symptoms or altered consciousness (0.50 months) and longest for dizziness (1.10 months).

Median time to diagnosis appeared to vary with age and tumor type, but not gender (Supplementary Table A11). For embryonal, cranial nerves, meningiomas and other mesenchymal tumors, time from earliest symptoms to diagnosis decreased with age, while it increased with age for other astrocytic tumors. Analyses by topography suggest that time from earliest symptom to diagnosis decreased with age for cerebellum, parietal lobes and overlapping lesion of the brain. The longest time to diagnosis was for brainstem and occipital lobe in the older age group. In terms of time from the latest symptoms, neuronal and mixed neuronal-glial tumors had the longest time to diagnosis.

Analyses of time between the first image that showed a space-occupying lesion and diagnosis showed a median difference of about one week (Supplementary Table A15). For the vast majority of morphologies, cases were diagnosed within 1 month, though the delay was one year or more in 1% of cases; these showed no specific characteristics: median age 15.5 years; no difference by gender; main symptoms: headaches (n = 5), nausea/vomiting (n = 3), visual sign and symptoms (n = 6), focal neurological signs and symptoms (n = 6) and convulsions (n = 3).

## Discussion

MOBI-Kids is the largest case–control study of BT in young people conducted so far, allowing investigation of the clinical characteristics of a large number of BT cases from 14 countries.

We found that gliomas were the most frequent tumors type, followed by embryonal tumors, as reported in previous publications [5, 9]. For topography, the most frequent locations were cerebellum and frontal lobe, as reported in other studies in young adults [9, 10]. As expected from the literature [11, 12], we found a significant decreasing trend with increasing age for embryonal tumors, and an increasing trend with age for meningioma and other non-neuroepithelial tumors. We also found a significant increasing trend with age for frontal and cerebral meninges tumors, but a decreasing trend for brainstem and cerebellum tumors, similar to previous publications [5].

There was a significant difference in the proportion of embryonal tumors by sex (sex ratio—male/female—2.31) which merits further study.

Similar to results of previous meta-analyses [13–15] and other studies [9, 16–20], we found a high prevalence of headache, focal neurological signs and symptoms, nausea/vomiting and visual signs and symptoms among our BT cases.

There were significant differences for all symptoms across topographies. Headaches and nausea/vomiting were most frequently reported for posterior fossa tumors as reported by others [15]. Visual signs and symptoms were most frequently reported for cerebral meninges, occipital lobe, and cranial nerves locations, in accordance with the location of the visual cortex and pathways of optical nerve. Interestingly, such symptoms were also frequent in cases with tumors in the cerebral ventricles and infratentorial locations, which may be explained by proximity to visual pathways or cranial nerve nuclei.

The patterns of symptoms, depending on tumor location, grade and morphology, demonstrates a remarkable consistency across studies although the levels of prevalence may differ. We found repeatedly headaches followed by nausea and vomiting as the most frequent symptoms, as reported by others [9, 18]. Intracranial pressure (ICP) is considered the cause of the joint occurrence of headaches and vomiting (without nausea). In general, variations in symptoms prevalence may be related to differences in ICP due to the growing tumor mass [10, 18, 21] that can exert pressure not only where the tumor is located but also in other areas.

The widespread use and accessibility of imaging techniques has contributed to earlier diagnosis of CNS tumors but diagnostic latencies are still sometimes of several months [9, 16, 18–25]; there is in fact even evidence that it not been shortened in the past decades [26]. As diagnostic latency increases, the tumor can grow or spread and eventually may lead to increased case fatality and/or long-term neurological and psychological sequelae (depending on the growth characteristics and aggressiveness of the tumor) [27, 28]. It has been suggested that behavioral symptoms (in medulloblastoma) are related with a longer diagnostic delay [29] and it is possible that behavioral changes in teenagers may, at first, be attributed to puberty [19, 30]. Our results partly confirm this observation, with cognitive, memory and behavioral changes having the longest latency among all symptoms in embryonal tumors, except for rare occurrences of seizures. Though it has been suggested that older children tend to have longer diagnostic delays than younger ones [22], we found no differences between age groups.

The majority of cases were diagnosed within 2 months after first symptom, despite the non-specificity of most signs and symptoms, consistent with earlier findings [17, 23, 31–33]. Longer latencies were found in 12% of cases, in line with a previous study [21]. There was little difference in symptom prevalence between latency groups, suggesting that the nature of symptoms does not greatly influence diagnostic latency. The median number of symptoms per case was 2, with some indication of differences by age and gender. Girls may communicate more freely about their symptoms with parents and health-care providers and parents are more aware of symptoms among children than among adolescents and young adults. Because malignant tumors grow faster than benign tumors, they generally lead to increase in ICP earlier, which affects occurrence of symptoms [16, 17, 20]. We found that children with high-grade tumors had a higher number of symptoms and an earlier diagnosis than those with low-grade tumors. Other studies suggested a positive correlation between vomiting and ataxia/gait abnormality and tumor grade, with these symptoms being more frequent in high grade tumors [19]. A major challenge in the diagnosis of BTs is that symptoms are often unspecific. Nevertheless, the joint occurrence of multiple symptoms or signs, or their persistent presence should generate awareness of the possibility of BT. Even if an underlying tumor is unlikely, patients and/or care-givers should be encouraged to contact health-care providers again should symptoms persist or progress. According to our results, these symptoms are more frequent for tumors with grade IV but also for grade I, which does not totally agree with earlier findings.

Our study has some limitations. Signs and symptoms were extracted from the clinical documentation of the cases and accuracy of these data depends on the completeness and accurateness of documentation, on the history given by patients and parents/caretakers and the signs detected by the examining physician. However, medical decisions will always be based on patients’ histories and findings obtained by examination of the patient rather than by an ideal and full spectrum of facts. We assumed that signs or symptoms not mentioned in the clinical record did not occur. There was no central review of radiology and histopathology although validation studies are under way. Concerning topography, we used up to three sources of evidence: the radiologist’s report, the surgery report, and the tumor mapped in a 3-D brain model (XGridMaster) by neuroradiologists. In case of discrepancy, we contacted the regional coordinator who checked, as necessary, with the treating physician. Concerning morphologies, we assigned broad categories that are likely unaffected by slight differences in neuropathologists’ categorizations.

Another limitation relates to the exclusion criteria the MOBI-Kids study applied, since tumors located in the midline area were excluded, thereby excluding pituitary tumors that are quite frequent in this age range and preventing us from providing information on these tumors. Further, limiting the age eligibility range to 10–24 years was a pragmatic solution, and not physiology-based since there is no distinct cut-off at 10 years concerning BT morphology and topography. The upper age of 24 years can arguably be defended by pointing to the well-known transition of BT types around this age. Because we excluded children below 10 years of age, our results are less affected by age-related difficulties to identify and specify symptoms [34]. Another limitation is the omission of information about the time of day symptoms occurred (a shortcoming for headaches that, when occurring in the morning, are considered to be more likely related to BTs).

The main strength is the fact that data were collected using a common protocol in 14 countries around the world. Clinical information was collected shortly after diagnosis and all cases were histologically confirmed. To our knowledge, no other study to date included so many cases in young people, for which data on clinical appearance are largely lacking.

## Conclusion

MOBI-Kids is the largest study of BT in young people conducted so far and it provided the opportunity to study signs, symptoms and other clinical characteristics of BT in young people. Many signs and symptoms were identified, dominated by headaches and nausea/vomiting. We also provided comprehensive information for very rare tumor types to provide input for future reviews.

Though the vast majority of tumors were diagnosed rapidly, within two months from first reported symptoms, 12% of cases were not diagnosed until at least one year after symptoms. Further studies of the characteristics of tumors with long times to diagnosis may ensure faster access to treatment.

## Electronic supplementary material

Below is the link to the electronic supplementary material.

Supplementary file1 (DOCX 479 kb)
