# Supplementary material for: Clinical presentation of young people (10–24 years old) with brain tumors: results from the international MOBI-Kids study
Source: J Neurooncol. 2020 Mar 3;147(2):427–40. doi: 10.1007/s11060-020-03437-4 (PMC7136306; doi:10.1007/s11060-020-03437-4)
Supplement: Supplementary file 1 — Supplementary file1 (DOCX 479 kb) [file 11060_2020_3437_MOESM1_ESM.docx]

**Supplementary material**

# Clinical presentation of young people (10-24 years old) with brain tumors: Results from the international MOBI-Kids study

*Angela Zumel-Marne ^1,2,3^;* *Michael Kundi ^4^; Gemma Castano-Vinyals ^1,2,3,5^; Juan Alguacil* ^3,6^; Eleni Petridou ^7,8^; Marios K. Georgakis ^7^; Maria Morales Suárez-Varela ^3,9^; Siegal Sadetzki ^10,11^; Sara Piro ^12^; Rajini Nagrani ^13,14^; Graziella Filippini ^15^; Hans-Peter Hutter ^4^; Rajesh Dikshit ^13^; Adelheid Woehrer ^16^_;_ Milena Maule ^17^; Tobias Weinmann ^18^; Daniel Krewski ^19^; Andrea ’t Mannetje ^20^; Franco Momoli ^18,21,22^; Brigitte Lacour ^23,24^; Stefano Mattioli ^25^; John Spinelli ^26^; Paul Ritvo ^27^*; *Thomas Remen ^22^; Noriko Kojimahara ^28^; Amanda Eng ^19^; Angela Thurston ^19^; Hyung-ryul Lim ^29^; Mina Ha ^29^; Naohito Yamaguchi ^28^; Charmaine Mohipp ^30,31^; Evdoxia Bouka ^11^; Chelsea Eastman ^1,2,3^; Roel Vermeulen ^32^; Hans Kromhout ^33^; Elisabeth Cardis ^1,2,3^.*

*^1^ ISGlobal, Barcelona, Spain; ^2^ Universitat Pompeu Fabra (UPF), Barcelona, Spain;^3^ Ciber Epidemiología y Salud Pública (CIBERESP), Madrid, Spain;^4^ Center for Public Health, Department of Environmental Health, Medical University Vienna, Vienna, Austria; ^5^ IMIM (Hospital del Mar Medical Research Institute), Barcelona, Spain; ^6^ Centro de Investigación en Salud y Medio Ambiente (CYSMA), Universidad de Huelva, Huelva, Spain; ^7^ Department of Hygiene, Epidemiology and Medical Statistics, Medical School, National and Kapodistrian University of Athens, Athens, Greece; ^8^ Clinical Epidemiology Unit Karolinska Institute, Stockholm,* *Sweden; ^9^ Área de Medicina Preventiva y Salud Pública, Universitat de Valencia, Valencia, Spain; ^10^ Cancer and Radiation Epidemiology Unit, Gertner Institute, Chaim Sheba Medical Center, Ramat Gan, Israel; ^11^ Sackler Faculty of Medicine, Tel Aviv University, Tel Aviv, Israel; ^12^ Environmental and Occupational Epidemiology Branch, Cancer Risk Factors and Lifestyle Epidemiology Unit, Institute for Cancer Research Prevention and Clinical Network-ISPRO, Florence, Italy;^13^ Centre for Cancer Epidemiology, Tata Memorial Centre, Mumbai, India; ^14^ Leibniz Institute for Prevention Research and Epidemiology - BIPS, Bremen, Germany; ^15^ Scientific Director’s Office, Fondazione IRCCS Istituto Neurologico Carlo Besta, Milano, Italy; ^16^ Institute of Neurology, Medical University Vienna, Vienna, Austria; ^17^ Unit of Cancer Epidemiology, Department of Medical Sciences, University of Turin, Turin, Italy; ^18^ Institute and Clinic for Occupational, Social and Environmental Medicine, University Hospital, LMU Munich, Munich, Germany; ^19^ School of Epidemiology and Public Health, University of Ottawa, Canada; ^20^ Centre for Public Health Research, Massey University,Wellington, New Zealand; ^21^ Ottawa Hospital Research Institute, Ottawa, ON, Canada; ^22^ Children’s Hospital of Eastern Ontario Research Institute, Ottawa, ON, Canada; ^23^ French National Registry of Childhood Solid Tumors, CHU, Nancy, France; ^24^ Inserm, Center of Research in Epidemiology and StatisticS (CRESS), Paris University, Epidemiology of childhood and adolescent cancers team (EPICEA), Paris, France; ^25^ Department of Medical and Surgical Sciences (DIMEC), University of Bologna, Bologna, Italy; ^26^ Population Oncology, BC Cancer, Vancouver, Canada, and School of Population and Public Health, University of British Columbia, Vancouver, Canada; ^27^ School of Kinesiology and Health Science and Department of Psychology at York University, Toronto, Canada; ^28^ Department of Public Health, Tokyo Women’s Medical University, Tokyo, Japan; ^29^ Department of Preventive Medicine, Dankook University College of Medicine, Cheonan, South Korea; ^30^ Children’s Hospital of Eastern Ontario, Ottawa, ON, Canada; ^31^ University of Ottawa, Ottawa, ON, Canada; ^32^ Division Environmental Epidemiology, Institute for Risk Assessment Sciences, Utrecht University, Utrecht, Netherlands; ^33^ Division Environmental Epidemiology, Institute for Risk Assessment Sciences, Utrecht University, Utrecht, Netherlands*

***Correspondence**: Prof. Dr. Juan Alguacil

Environmental Epidemiology and Neuroscience Group

Universidad de Huelva

Av. Andalucía s/n

21071 Huelva, Spain

Tel: +34- 959219890

E-mail: alguacil@dbasp.uhu.es

**Additional information about material and methods**

# Main variables: morphology, topography, laterality, signs and symptoms

## Morphology:

The information about morphology was coded using either the 1^st^ or 3^rd^ revision of the ICD-O (International Classification of Disease for Oncology), depending on the time period and hospital, and complemented by the diagnosis in plain text. The questionnaire allowed entering up to three morphology codes, as well as information about diagnosis when a second opinion was sought.

Morphologies were grouped according to the 2016 World Health Organization Classification of Tumors of the Central Nervous System [1]. Detailed categories are available in Supplementary Table A1.

## Location of tumor:

### Anatomical location:

Anatomical location of the tumor in the head was collected based on a pre-defined list of locations. Depending on the presence of multifocal tumors and the size, tumors might be assigned to more than one anatomical location. For cases with a tumor in more than two locations or those without a specific location in the cerebrum, morphology and topography were checked on a case by case basis in order to define the location occupying the largest volume of the tumor. Some tumors spreading through several regions of the brain were categorized as overlapping.

### Topography:

The setup of the clinical questionnaire for topography was similar to morphology. Up to three ICD codes for topography together with plain text of location were provided. The topographic categories were selected according to those typically used in the literature [1] and the prevalence observed in the study. An ‘other location’ category was created to include all other areas with less specific location such as: brain unspecific, and other parts of the brain, and brain NOS (not otherwise specified).

### Laterality:

We collected information regarding the side of the head where the tumor was located. This information was reclassified into: left (including both sides, but major left; both central and left), right (including both sides, but major right; both central and right), central and no assignment. This information was also used to compare with lateralized signs and symptoms into the following categories: ipsilateral, contralateral, no side assigned, and unknown. Ipsilateral was assigned if the tumor location was on the same side as the reported symptom; contralateral was assigned if the tumor was on the opposite of the side specified for a sign or symptom reported. The category of ‘no side assigned’ was used if the symptom had no localization (e.g., vomiting) or if the information was unavailable.

## Grade of tumors:

We used the WHO classification [1] for the grading of tumors (grade I to IV); further classified as low grade tumors (grades I and II) and high grade tumors (grades III and IV).

## Signs and symptoms:

Information about signs and symptoms, reported before surgery, was collected in the clinical questionnaire (except for cases of Austria and France). For convenience, henceforth in the paper ‘symptoms’ will refer to both: signs and symptoms. These symptoms could be experienced by the patient or determined by the clinician in a physical exam. The first part of the questionnaire about symptoms listed fourteen categories: headaches, dizziness, vomiting/nausea, general weakness, general deterioration, loss of consciousness, decreased level of consciousness, behavioral changes/confusion, cognitive changes, memory disturbances, convulsions, dysphasia, ataxia/imbalance, and tremors. The second part listed five specific items with (possibly) lateralized symptoms (right, left, both sides): limb weakness, hemiparesis, hypoesthesia, vision disturbance, and papilledema with an additional item to indicate whether the symptom was reported by the patient or by the doctor during a clinical examination. Additional symptoms were allowed to be entered in open text fields.

All symptoms were re-classified based on the guideline of the Children’s Brain Tumor Research Centre at Nottingham University [2]. Overall, nine main categories were chosen: headaches; nausea/vomiting; visual signs and symptoms; focal neurological signs and symptoms (which appended the Guidelines’ ‘motor symptoms and signs’ category); cognitive, memory and behavioral changes; convulsions/seizures; altered consciousness; dizziness; altered sensitivity. Study records included more than 75 specific symptoms (Supplementary Table A2). Symptoms classified as focal neurological signs and symptoms were those which are often ascribed to certain regions of the brain although there were overlaps (e.g. visual signs and symptoms have been chosen as a distinct category because of their frequency but could also be classified into focal neurological signs and symptoms).

# Calculation of the time between the first symptom and diagnosis:

The date of final confirmation of a diagnosis was defined as the date of biopsy, or, if this was not available, the date of surgery. When no biopsy or surgery was conducted, then the date of the first unequivocal imaging (by CT scan and/or MRI) was used. For each symptom, the number of days between the reported first occurrence of the symptom and the date of final confirmation of diagnosis was calculated. Some symptoms were reported after the diagnosis date and were not taken into account in the analyses.

We categorized the time between symptoms and diagnosis in the following way: less than 1 month, equal to 1 and up to 2 months, equal to 2 months and up to 6 months, equal to 6 months to up to 1 year, equal to 1 year and up to 2 years, and more than 2 years.

Additionally, we created one variable called "earliest symptom" that represents the first symptom that was reported before diagnosis, and another one, "latest symptom", that represents the symptom closest to diagnosis.

# Categorization of other variables: age

Age of the cases was categorized into three groups: 10 to 14, 15 to 19, and 20 to 24 years old, based on the incidence of BTs and previous publications [3–8].

# Statistical analyses

We calculated the median and interquartile range of timing (in months) between date of first symptom until date of final diagnosis (information available for 636 cases). We also calculated the median number of symptoms reported by each case and the time between last symptom and diagnosis dates.

We conducted descriptive analyses of information collected, including: tests of heterogeneity of the distribution of morphology and topography by sex, age and grade of tumor using chi-square tests [9]; and tests for differences in time (between symptoms and diagnosis) by topography, morphology, age, sex and grade of tumor using Kruskal–Wallis tests [10].

We performed cluster analyses across symptoms using Dice dissimilarity coefficients (DDC) using complete linkage as the amalgamation rule. This was done for all available symptomatic cases, and for the main morphological and topographical categories (Supplementary Figure A3-A5).

Regression analysis of time-to-diagnosis (from earliest symptom to date of diagnosis) on gender, age, morphology and topography was performed log-transformation of time to diagnosis applying the general linear model.

All analyses were performed using Stata v.14 (StataCorp, TX, USA).

# References

1. Louis DN, Perry A, Reifenberger G, et al (2016) The 2016 World Health Organization Classification of Tumors of the Central Nervous System: a summary. Acta Neuropathol (Berl) 131:803–820. https://doi.org/10.1007/s00401-016-1545-1

2. Wilne S, Koller K, Collier J, et al (2010) The diagnosis of brain tumours in children: a guideline to assist healthcare professionals in the assessment of children who may have a brain tumour. Arch Child 95:534–9. https://doi.org/10.1136/adc.2009.162057

3. Dang-Tan T, Trottier H, Mery LS, et al (2008) Delays in diagnosis and treatment among children and adolescents with cancer in Canada. Pediatr Blood Cancer 51:468–74. https://doi.org/10.1002/pbc.21600

4. Johannesen TB, Angell-Andersen E, Tretli S, et al (2004) Trends in incidence of brain and central nervous system tumors in Norway, 1970-1999. Neuroepidemiology 23:101–9. https://doi.org/10.1159/000075952

5. McKinney PA (2004) Brain tumours: incidence, survival, and aetiology. J Neurol Neurosurg Psychiatry 75 Suppl 2:ii12-7

6. Turner MC, Gracia-Lavedan E, Momoli F, et al (2019) Nonparticipation Selection Bias in the MOBI-Kids Study. Epidemiol Camb Mass 30:145–153. https://doi.org/10.1097/EDE.0000000000000932

7. Rémen T, Lacour B (2018) [Use of wireless telecommunications technologies among the 10-25-year-old in France: Data extracted from the French part of the MOBI-KIDS study]. Rev Epidemiol Sante Publique. https://doi.org/10.1016/j.respe.2018.04.058

8. Sadetzki S, Langer CE, Bruchim R, et al (2014) The MOBI-Kids Study Protocol: Challenges in Assessing Childhood and Adolescent Exposure to Electromagnetic Fields from Wireless Telecommunication Technologies and Possible Association with Brain Tumor Risk. Front Public Health 2:. https://doi.org/10.3389/fpubh.2014.00124

9. McHugh ML (2013) The chi-square test of independence. Biochem Medica 23:143–149

10. Theodorsson-Norheim E (1986) Kruskal-Wallis test: BASIC computer program to perform nonparametric one-way analysis of variance and multiple comparisons on ranks of several independent samples. Comput Methods Programs Biomed 23:57–62

**Figure A1. Distribution of cases by main tumor locations.**

**
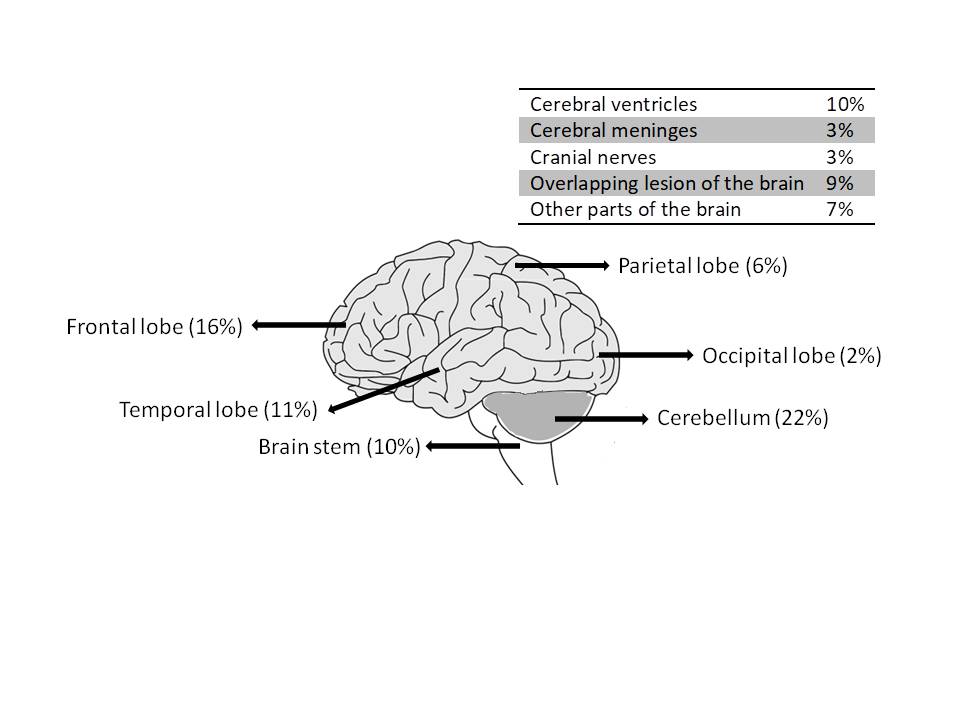
**

**Figure A2.** **Symptoms by topography**.

Frontal lobe:

Temporal lobe:


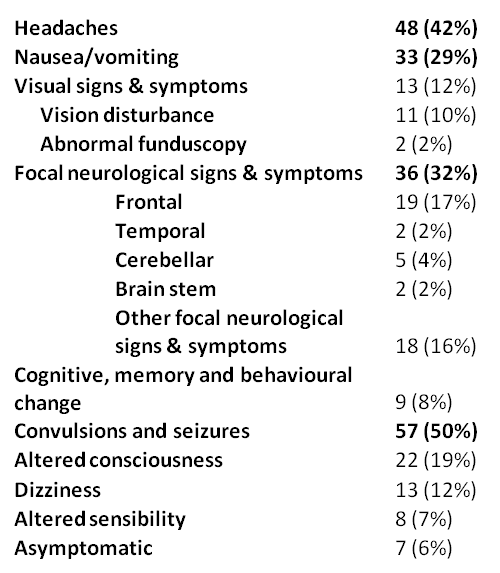

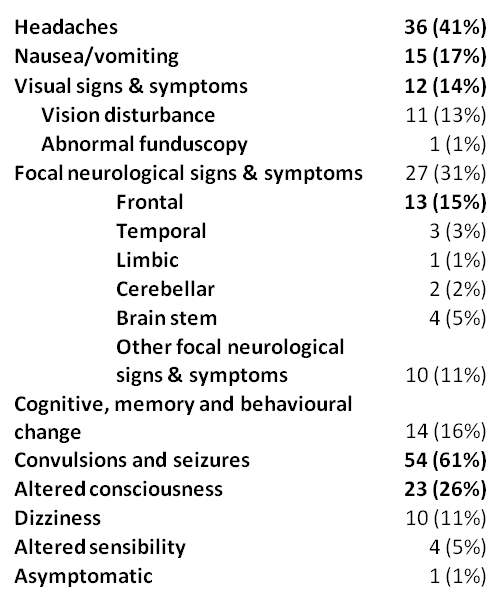

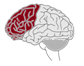

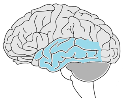


Occipital lobe:


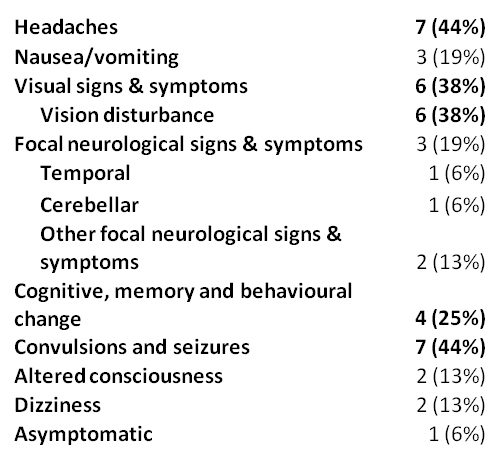


Parietal lobe:


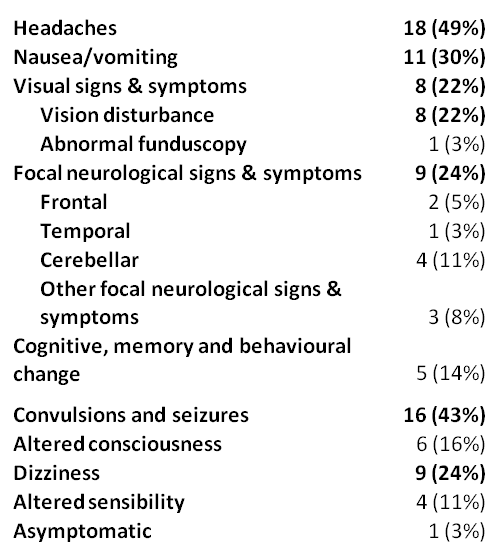

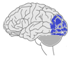

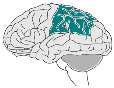


Cerebellum:


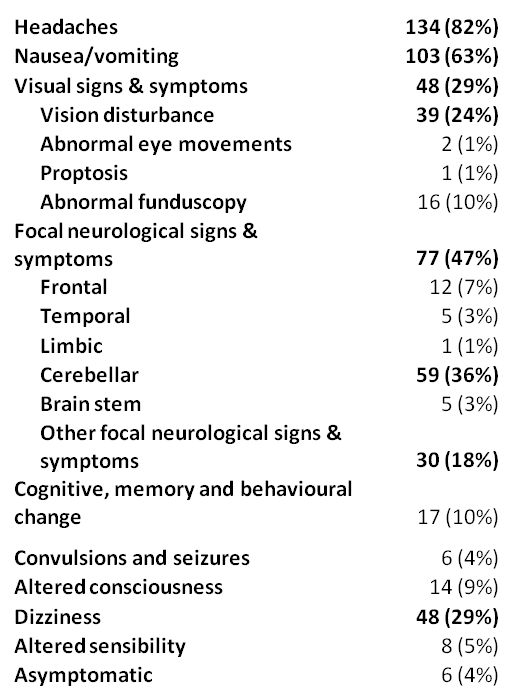

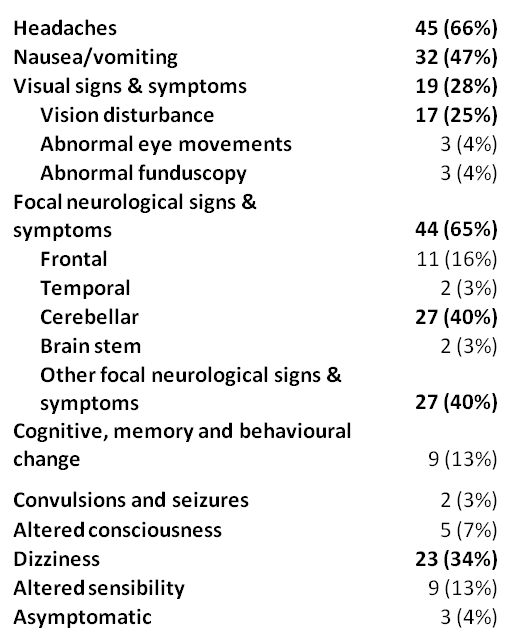


Brain stem:


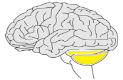

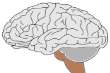


**Figure A3.** Cluster analysis of symptoms preceding brain tumor diagnosis for all cases (n=722) with information on symptoms based on Dice dissimilarity metric and complete linkage (Abbreviations: H...headache, N/V...nausea/vomiting, Focal...focal neurological signs & symptoms, Vis...visual signs & symptoms, Cog...cognitive, memory & behavioral change, Diz...Dizziness, Sens...altered sensibility, Seiz...convulsions/seizures, Cons...altered consciousness).

**Figure A4.** Cluster analysis of symptoms preceding brain tumor diagnosis for cases with neuroepithelial tumors (n=551) (A); gliomas (n=459) (B); 'other neuroepithelial' tumors (n=92) (C); embryonal tumors (n=102) (D); meningioma tumors (n=35) (E); 'other non-neuroepithelial' tumors (n=34) (F) with information on symptoms based on Dice dissimilarity metric and complete linkage (Abbreviations: H...headache, N/V...nausea/vomiting, Focal...focal neurological signs & symptoms, Vis...visual signs & symptoms, Cog...cognitive, memory & behavioral change, Diz...Dizziness, Sens...altered sensibility, Seiz...convulsions/seizures, Cons...altered consciousness)

**Figure A5.** Cluster analysis of symptoms preceding brain tumor diagnosis for cases with tumors on brainstem (n=68) (A); cerebellum (n=163) (B); frontal lobe (n=113) (C); temporal lobe (n=88); parietal lobe (n=37) (D); occipital lobe (n=16) (E); 'other parts of the brain' (n=237) (F) with information on symptoms based on Dice dissimilarity metric and complete linkage (Abbreviations: H...headache, N/V...nausea/vomiting, Focal...focal neurological signs & symptoms, Vis...visual signs & symptoms, Cog...cognitive, memory & behavioral change, Diz...Dizziness, Sens...altered sensibility, Seiz...convulsions/seizures, Cons...altered consciousness).

**Table A1. ICD codes and morphology groups**

| **Morphology** | | | |
| --- | --- | --- | --- |
| **ICD code** | **Type** | **Subgroup** | **Group** |
| 9380/3; 9381/3; 9382/3; 9383/1; 9384/1; 9391/3; 9392/3; 9393/3; 9400/3; 9394/1; 9401/3; 9410/3; 9411/3; 9420/3; 9421/1; 9424/3; 9425/3; 9440/3; 9441/3; 9442/1; 9442/3; 9450/3; 9451/3; 9460/3 | Glioma | Glioma | Neuroepithelial |
| 8680/0; 8680/1; 8680/3; 8690/1; 9412/1; 9413/0; 9492/0; 9493/0; 9505/1; 9505/3; 9506/1; 9509/1 | Neuronal and mixed | Other neuroepithelial |  |
| 9430/3; 9431/1; 9444/1 | Other neuroepithelial |  |  |
| 9470/3; 9471/3; 9472/3; 9473/3; 9474/3; 9480/3; 9490/3; 9500/3; 9501/3 | Embryonal | Embryonal | Non neuroepithelial |
| 9530/0; 9530/1; 9530/0-1; 9530/3; 9531/0; 9532/0; 9533/0; 9534/0; 9535/0; 9537/0; 9538/1; 9538/3; 9539/1; 9539/3 | Meninges | Meninges |  |
| 9540/0; 9540/1; 9540/0-1; 9540/3; 9550/0; 9560/0; 9560/1; 9560/0-1; 9571/0; 9571/3 | Cranial paraspinal nerves | Other non neuroepithelial |  |
| 9390/0; 9390/1; 9390/3 | Choroid plexus |  |  |
| 9150/1; 9161/1 | Other non neuroepithelial |  |  |

**Table A2. Description of detailed symptoms included in each category**

| **Main category** | **Subcategory** | **Included…** |
| --- | --- | --- |
| Headaches |  | Headaches |
| Nausea/vomiting |  | Nausea |
|  |  | Vomiting |
|  |  | Emesis salivary |
|  |  | Vegetative accompanying symptoms |
| Visual signs & symptoms |  |  |
|  | Vision disturbance | Vision disturbance |
|  | Abnormal eye movements | Nystagmus |
|  |  | Weakness of muscle orbicularis oculi |
|  | Proptosis | Proptosis |
|  | Abnormal funduscopy | Papillary hemorrhage |
|  |  | Papilledema |
| Focal neurological signs & symptoms |  |  |
|  | Frontal | Paresis |
|  |  | Dysphasia |
|  |  | Hemiparesis |
|  |  | Paralysis |
|  |  | Newborn reflexes |
|  |  | Babinski reflex positive |
|  |  | Susp. Babinski reflex positive |
|  |  | Hoffmann's reflex positive |
|  |  | Anosmia |
|  | Temporal | Tinnitus |
|  |  | Sound hallucinations |
|  |  | Hearing problems |
|  |  | Hearing loss |
|  | Limbic | Olfactory dysfunction |
|  |  | Anorexia |
|  |  | Recently developed spells of smells. The smells are of something funny |
|  | Cerebellar | Ataxia/imbalance |
|  |  | Right pronator drift with 4+/5 power UL 4-/5 lower limb |
|  |  | Intention tremor |
|  | Brainstem | Photo- and phonophobia |
|  |  | Decreased reaction of pupil to light |
|  |  | Dry eyes and mouth |
|  |  | Photophobia |
|  |  | Phonophobia |
|  |  | Drooling |
|  |  | Sonophobia |
|  |  | Acoustic hypersensitivity |
|  |  | Excessive noise sensitivity ear |
|  |  | Hiccups / globus sensation |
|  |  | Photosensitivity |
|  |  | Sonophobia |
|  |  | Abnormal gait |
|  | Other | Swallowing problems |
|  |  | Dysphagia |
|  |  | Difficulty in swallowing of liquids |
|  |  | Tremor |
|  |  | Limb weakness |
|  |  | Weakness |
|  |  | Neurogenic bladder dysfunction |
|  |  | Diabetes insipidus |
|  |  | Bradydiadochokinese |
|  |  | Deficit coordination |
|  |  | Dysdiadochokinesia |
|  |  | Minimum Dsc diadochokinesis |
|  |  | Loss of lock arm |
|  |  | Mouth floor contractions |
|  |  | Deviation of mimicry muscles and head |
|  |  | Troemner reflex positive |
|  |  | Hyperreflexia |
|  |  | increasing of leg reflexes |
| Cognitive, memory and behavioral changes |  | Cognitive, memory and behavioral changes |
| Convulsions/seizures |  | Convulsions |
|  |  | Petit-mal seizure |
|  |  | Epilepsy |
|  |  | Seizures |
| Altered consciousness |  | Decreased or loss of consciousness |
|  |  | Hypersomnia |
| Dizziness |  | Dizziness |
| Altered sensibility |  | Hypoesthesia |
|  |  | Feeling cold |
|  |  | Sensory disturbance instep / plantar |
|  |  | Tingle |
|  |  | Several types of body pain |
|  |  | General discomfort |
|  |  | Arthromyalgia |

**Table A3. Morphology by topography**

|  | **Topography** | | | | | | | | | | | | |
| --- | --- | --- | --- | --- | --- | --- | --- | --- | --- | --- | --- | --- | --- |
| **Morphology:** | **Total (N=899)** | **Brainstem (n=91)** | **Cerebellum (n=201)** | **Frontal lobe (n=143)** | **Temporal lobe (n=103)** | **Parietal lobe (n=52)** | **Occipital lobe (n=17)** | **Cerebral ventricles (n=88)** | **Cerebral meninges (n=31)** | **Cranial nerves (n=30)** | **Overlapping lesion of the brain (n=83)** | **Other parts of brain** ^b^ **(n=60)** | ***p-value***^a^ |
| ***Neuroepithelial:*** | *676* | *81 (12%)* | *104 (16%)* | *131 (19%)* | *96 (14%)* | *49 (7%)* | *15 (2%)* | *59 (9%)* | *1 (0%)* | *12 (2%)* | *70 (10%)* | *58 (9%)* |  |
| Glioma | 556 | 77 (14%) | 95 (17%) | 112 (20%) | 57 (10%) | 34 (6%) | 12 (2%) | 39 (7%) | 0 (0%) | 12 (2%) | 64 (12%) | 54 (10%) | <0.05 |
| Glioma- High grade^c^ | 201 | 39 (20%) | 14 (7%) | 52 (26%) | 15 (7%) | 12 (6%) | 2 (1%) | 13 (6%) | 0 (0%) | 6 (3%) | 20 (10%) | 28 (14%) | <0.05 |
| Glioma- Low grade^c^ | 355 | 38 (11%) | 81 (23%) | 60 (17%) | 42 (12%) | 22 (6%) | 10 (3%) | 26 (7%) | 0 (0%) | 6 (2%) | 44 (12%) | 26 (7%) |  |
| Other neuroepithelial | 120 | 4 (3%) | 9 (7%) | 19 (16%) | 39 (32%) | 15 (13%) | 3 (3%) | 20 (17%) | 1 (1%) | 0 (0%) | 6 (5%) | 4 (3%) |  |
| ***Non neuroepithelial:*** | *223* | *10 (5%)* | *97 (44%)* | *12 (5%)* | *7 (3%)* | *3 (1%)* | *2 (1%)* | *29 (13%)* | *30 (13%)* | *18 (8%)* | *13 (6%)* | *2 (1%)* |  |
| Embryonal tumors | 129 | 9 (7%) | 86 (67%) | 6 (5%) | 2 (1%) | 1 (1%) | 2 (1%) | 11 (8%) | 0 (0%) | 0 (0%) | 10 (8%) | 2 (2%) | <0.05 |
| Meningiomas | 47 | 0 (0%) | 1 (2%) | 6 (13%) | 3 (6%) | 1 (2%) | 0 (0%) | 4 (9%) | 30 (64%) | 1 (2%) | 1 (2%) | 0 (0%) |  |
| Other non neuroepithelial | 47 | 1 (2%) | 10 (21%) | 0 (0%) | 2 (4%) | 1 (2%) | 0 (0%) | 14 (31%) | 0 (0%) | 17 (36%) | 2 (4%) | 0 (0%) |  |

^a^p-values chi2 for heterogeneity; percentage by row; ^b^ including brain NOS; ^c^High grade (grade I-II) and Low grade (grade III-IV).

**Table A4. Morphology and topography by grade**

|  | **Grade of tumor (WHO)** | | | | |  |
| --- | --- | --- | --- | --- | --- | --- |
| **Morphology:** | ***Total (N=899)*** | **I  (n=386)** | **II  (n=189)** | **III  (n=130)** | **IV  (n=194)** | ***p-value*** ^a^ |
| ***Neuroepithelial:*** | *676* | *305 (45%)* | *165 (24%)* | *140 (21%)* | *66 (10%)* |  |
| **Glioma** | *556* | 208 (38%) | 147 (26%) | 139 (25%) | 62 (11%) | <0.05 |
| **Other neuroepithelial** | *120* | 97 (81%) | 18 (15%) | 1 (1%) | 4 (3%) |  |
| ***Non neuroepithelial*** | *223* | *81 (36%)* | *12 (5%)* | *2 (1%)* | *128 (58%)* |  |
| **Embryonal tumors** | *129* | 0 (0%) | 3 (2%) | 1 (1%) | 125 (97%) |  |
| **Meningiomas** | *47* | 36 (77%) | 10 (21%) | 1 (2%) | 0 (0%) | <0.05 |
| **Other non neuroepithelial** | *47* | 45 (96%) | 2 (4%) | 0 (0%) | 0 (0%) |  |
| **Topography:** |  |  |  |  |  |  |
| **Brainstem** | *91* | 26 (28%) | 17 (19%) | 31 (34%) | 17 (19%) |  |
| **Cerebellum** | *201* | 88 (44%) | 13 (6%) | 12 (6%) | 88 (44%) |  |
| **Frontal lobe** | *143* | 35 (25%) | 48 (34%) | 30 (21%) | 30 (21%) |  |
| **Temporal lobe** | *103* | 62 (60%) | 23 (22%) | 9 (9%) | 9 (9%) |  |
| **Parietal lobe** | *52* | 24 (46%) | 15 (29%) | 9 (17%) | 4 (8%) |  |
| **Occipital lobe** | *17* | 7 (41%) | 6 (35%) | 1 (6%) | 3 (18%) | <0.05 |
| **Cerebral ventricles** | *88* | 38 (43%) | 25 (28%) | 11 (12%) | 14 (16%) |  |
| **Cerebral meninges** | *31* | 22 (71%) | 8 (26%) | 1 (3%) | 0 (0%) |  |
| **Cranial nerves** | *30* | 23 (77%) | 1 (3%) | 6 (20%) | 0 (0%) |  |
| **Overlapping lesion of the brain** | *83* | 39 (47%) | 13 (16%) | 15 (18%) | 16 (19%) |  |
| **Other parts of the brain** ^b^ | *60* | 22 (37%) | 8 (13%) | 17 (28%) | 13 (22%) |  |
|  |  |  |  |  |  |  |
|  |  |  |  |  |  |  |

^a^ p-value by chi2; percentage by row, based in all cases; ^b^ Including brain NOS .

**Table A5. Detailed symptoms by morphology**

|  |  |  |  | | **Morphology** | | | | | | | |  |
| --- | --- | --- | --- | --- | --- | --- | --- | --- | --- | --- | --- | --- | --- |
|  |  |  |  | | **Neuroepithelial** | | | **Non Neuroepithelial** | | | | |  |
|  | ***Total cases with symptoms information (n=722)*** | **All gliomas (n=459)** | **Glioma- high grade**  **(n=163)^b^** | **Glioma- low grade**  **(n=296)^b^** | | **Neuronal and mixed (n=89)** | **Other neuroepithelial (n= 3)** | **Embryonal tumors (n=102)** | **Meningiomas (n=35)** | **Cranial pasapinal nerves (n=14)** | **Choroid plexus tumors (n=12)** | **Other non neuroepithelial (n=8)** | **p-value** ^a^ |
| **Symptoms** | ***N*** | **N (%)** | **N (%)** | **N (%)** | | **N (%)** | **N (%)** | **N (%)** | **N (%)** | **N (%)** | **N (%)** | **N (%)** |  |
| **Headaches** | *436* | 280 (61%) | 105 (64%) | 175 (59%) | | 40 (45%) | 2 (67%) | 79 (77%) | 13 (37%) | 6 (43%) | 8 (67%) | 8 (100%) | <0.05 |
| **Nausea/vomiting** | *280* | 179 (39%) | 72 (44%) | 106 (36%) | | 16 (18%) | 1 (33%) | 71 (70%) | 3 (9%) | 1 (7%) | 4 (33%) | 5 (63%) | <0.05 |
| **Visual signs & symptoms** | *217* | 133 (29%) | 52 (32%) | 81 (27%) | | 19 (21%) | 0 (0%) | 39 (38%) | 17 (49%) | 2 (14%) | 7 (58%) | 0 (0%) | <0.05 |
| **Vision disturbance** | *180* | 111 (24%) | 47 (29%) | 64 (22%) | | 18 (20%) | 0 (0%) | 30 (29%) | 14 (40%) | 2 (14%) | 5 (42%) | 0 (0%) | 0.08 |
| **Abnormal eye movements** | *15* | 9 (2%) | 5 (3%) | 4 (1%) | | 0 (0%) | 0 (0%) | 4 (4%) | 0 (0%) | 0 (0%) | 2 (17%) | 0 (0%) | 0.02 |
| **Proptosis** | *6* | 4 (1%) | 2 (1%) | 2 (1%) | | 0 (0%) | 0 (0%) | 0 (0%) | 2 (6%) | 0 (0%) | 0 (0%) | 0 (0%) | 0.10 |
| **Abnormal funduscopy** | *69* | 36 (8%) | 6 (4%) | 30 (10%) | | 4 (4%) | 0 (0%) | 19 (19%) | 5 (14%) | 1 (7%) | 4 (33%) | 0 (0%) | <0.05 |
| **Focal neurological signs & symptoms** | *287* | 185 (40%) | 68 (42%) | 117 (40%) | | 22 (25%) | 1 (33%) | 53 (52%) | 13 (37%) | 6 (43%) | 4 (33%) | 3 (38%) | 0.03 |
| **Frontal** | *87* | 61 (13%) | 20 (12%) | 41 (14%) | | 6 (7%) | 1 (33%) | 9 (9%) | 7 (20%) | 2 (14%) | 1 (8%) | 0 (0%) | 0.27 |
| **Temporal** | *23* | 11 (2%) | 4 (2%) | 7 (2%) | | 3 (3%) | 0 (0%) | 2 (2%) | 2 (6%) | 4 (29%) | 1 (8%) | 0 (0%) | <0.05 |
| **Limbic** | *4* | 0 (0%) | 0 (0%) | 0 (0%) | | 1 (1%) | 0 (0%) | 3 (3%) | 0 (0%) | 0 (0%) | 0 (0%) | 0 (0%) | 0.05 |
| **Cerebellar** | *136* | 89 (19%) | 34 (21%) | 55 (19%) | | 2 (2%) | 0 (0%) | 36 (35%) | 0 (0%) | 5 (36%) | 1 (8%) | 3 (38%) | <0.05 |
| **Brain stem** | *25* | 14 (3%) | 7 (4%) | 7 (2%) | | 4 (4%) | 0 (0%) | 5 (5%) | 1 (3%) | 1 (7%) | 0 (0%) | 0 (0%) | 0.92 |
| **Other focal neurological signs & symptoms** | *140* | 91 (20%) | 36 (22%) | 55 (16%) | | 10 (11%) | 1 (33%) | 29 (28%) | 4 (11%) | 2 (14%) | 2 (17%) | 1 (13%) | 0.12 |
| **Cognitive, memory and behavioral change** | *88* | 49 (11%) | 21 (13%) | 28 (9%) | | 15 (17%) | 0 (0%) | 17 (17%) | 2 (6%) | 1 (7%) | 2 (17%) | 2 (25%) | 0.31 |
| **Convulsions/seizures** | *174* | 106 (23%) | 32 (20%) | 74 (25%) | | 48 (54%) | 1 (33%) | 6 (6%) | 11 (31%) | 1 (7%) | 1 (8%) | 0 (0%) | <0.05 |
| **Altered consciousness** | *101* | 64 (14%) | 15 (9%) | 49 (17%) | | 22 (25%) | 0 (0%) | 10 (10%) | 2 (6%) | 1 (7%) | 1 (8%) | 1 (13%) | 0.06 |
| **Dizziness** | *141* | 88 (19%) | 39 (24%) | 49 (17%) | | 9 (10%) | 0 (0%) | 32 (31%) | 4 (11%) | 3 (21%) | 4 (33%) | 1 (13%) | 0.01 |
| **Altered sensibility** | *67* | 42 (9%) | 18 (11%) | 24 (8%) | | 9 (10%) | 0 (0%) | 6 (6%) | 3 (9%) | 6 (43%) | 1 (8%) | 0 (0%) | <0.05 |
| **Asymptomatic** | *27* | 16 (3%) | 3 (2%) | 13 (4%) | | 2 (2%) | 0 (0%) | 4 (4%) | 3 (9%) | 2 (14%) | 0 (0%) | 0 (0%) | 0.32 |

Percentage by column, based in cases with symptoms information; ^a^ p-value by chi2; ^b^High grade (grade I-II) and Low grade (grade III-IV).

**Table A6. Detailed symptoms by topography**

|  | **Topography** | | | | | | | | | | | | |
| --- | --- | --- | --- | --- | --- | --- | --- | --- | --- | --- | --- | --- | --- |
|  | ***Total cases with symptoms information (n=722)*** | **Brainstem (n=68)** | **Cerebellum (n=163)** | **Frontal lobe (n=113)** | **Temporal lobe (n=88)** | **Parietal lobe (n=37)** | **Occipital lobe (n=16)** | **Cerebral ventricles (n=74)** | **Cerebral meninges (n=24)** | **Cranial nerves (n=21)** | **Overlapping lesion of brain (n=76)** | **Other parts of brain** ^a^ **(n=42)** | **p value** ^b^ |
| **Symptoms** | ***N*** | **n (%)** | **n (%)** | **n (%)** | **n (%)** | **n (%)** | **n (%)** | **n (%)** | **n (%)** | **n (%)** | **n (%)** | **n (%)** |  |
| **Headaches** | *436* | 45 (66%) | 134 (82%) | 48 (42%) | 36 (41%) | 18 (49%) | 7 (44%) | 57 (77%) | 10 (42%) | 9 (43%) | 44 (58%) | 28 (67%) | <0.01 |
| **Nausea/vomiting** | *277* | 32 (47%) | 103 (63%) | 33 (29%) | 15 (17%) | 11 (30%) | 3 (19%) | 35 (47%) | 1 (4%) | 2 (10%) | 28 (37%) | 14 (33%) | <0.01 |
| **Visual signs & symptoms** | *217* | 19 (28%) | 48 (29%) | 13 (12%) | 12 (14%) | 8 (22%) | 6 (38%) | 37 (50%) | 16 (67%) | 9 (43%) | 30 (39%) | 19 (45%) | <0.01 |
| **Vision disturbance** | *180* | 17 (25%) | 39 (24%) | 11 (10%) | 11 (13%) | 8 (22%) | 6 (38%) | 27 (36%) | 14 (58%) | 8 (38%) | 22 (29%) | 17 (40%) | <0.01 |
| **Abnormal eye movements** | *15* | 3 (4%) | 2 (1%) | 0 (0%) | 0 (0%) | 0 (0%) | 0 (0%) | 5 (7%) | 0 (0%) | 0 (0%) | 5 (7%) | 0 (0%) | <0.01 |
| **Proptosis** | *6* | 0 (0%) | 1 (1%) | 0 (0%) | 0 (0%) | 0 (0%) | 0 (0%) | 0 (0%) | 2 (8%) | 1 (5%) | 1 (1%) | 1 (2%) | <0.01 |
| **Abnormal funduscopy** | *69* | 3 (4%) | 16 (10%) | 2 (2%) | 1 (1%) | 1 (3%) | 0 (0%) | 19 (26%) | 4 (17%) | 2 (10%) | 16 (21%) | 5 (12%) | <0.01 |
| **Focal neurological signs & symptoms** | *287* | 44 (65%) | 77 (47%) | 36 (32%) | 27 (31%) | 9 (24%) | 3 (19%) | 24 (32%) | 8 (33%) | 8 (38%) | 39 (51%) | 12 (29%) | <0.01 |
| **Frontal** | *87* | 11 (16%) | 12 (7%) | 19 (17%) | 13 (15%) | 2 (5%) | 0 (0%) | 6 (8%) | 4 (17%) | 2 (10%) | 12 (16%) | 6 (14%) | 0.17 |
| **Temporal** | *23* | 2 (3%) | 5 (3%) | 2 (2%) | 3 (3%) | 1 (3%) | 1 (6%) | 2 (3%) | 1 (4%) | 4 (19%) | 2 (3%) | 0 (0%) | 0.03 |
| **Limbic** | *4* | 0 (0%) | 1 (1%) | 0 (0%) | 1 (1%) | 0 (0%) | 0 (0%) | 1 (1%) | 0 (0%) | 0 (0%) | 1 (1%) | 0 (0%) | 0.95 |
| **Cerebellar** | *136* | 27 (40%) | 59 (36%) | 5 (4%) | 2 (2%) | 4 (11%) | 1 (6%) | 11 (15%) | 0 (0%) | 5 (24%) | 18 (24%) | 4 (10%) | <0.01 |
| **Brainstem** | *25* | 2 (3%) | 5 (3%) | 2 (2%) | 4 (5%) | 0 (0%) | 0 (0%) | 3 (4%) | 1 (4%) | 2 (10%) | 4 (5%) | 2 (5%) | 0.76 |
| **Other focal neurological signs & symptoms** | *140* | 27 (40%) | 30 (18%) | 18 (16%) | 10 (11%) | 3 (8%) | 2 (13%) | 12 (16%) | 3 (13%) | 3 (14%) | 28 (37%) | 4 (10%) | <0.01 |
| **Cognitive, memory and behavioral change** | *88* | 9 (13%) | 17 (10%) | 9 (8%) | 14 (16%) | 5 (14%) | 4 (25%) | 9 (12%) | 1 (4%) | 2 (10%) | 12 (16%) | 6 (14%) | 0.56 |
| **Convulsions/seizures** | *174* | 2 (3%) | 6 (4%) | 57 (50%) | 54 (61%) | 16 (43%) | 7 (44%) | 8 (11%) | 6 (25%) | 1 (5%) | 15 (20%) | 2 (5%) | <0.01 |
| **Altered consciousness** | *101* | 5 (7%) | 14 (9%) | 22 (19%) | 23 (26%) | 6 (16%) | 2 (13%) | 9 (12%) | 2 (8%) | 1 (5%) | 12 (16%) | 5 (12%) | 0.01 |
| **Dizziness** | *141* | 23 (34%) | 48 (29%) | 13 (12%) | 10 (11%) | 9 (24%) | 2 (13%) | 16 (22%) | 2 (8%) | 3 (14%) | 10 (13%) | 5 (12%) | <0.01 |
| **Altered sensibility** | *67* | 9 (13%) | 8 (5%) | 8 (7%) | 4 (5%) | 4 (11%) | 0 (0%) | 13 (18%) | 2 (8%) | 7 (33%) | 7 (9%) | 5 (12%) | <0.01 |
| **Asymptomatic** | *27* | 3 (4%) | 6 (4%) | 7 (6%) | 1 (1%) | 1 (3%) | 1 (6%) | 1 (1%) | 1 (4%) | 1 (5%) | 3 (4%) | 2 (5%) | 0.86 |

Percentage by column, based on cases with symptoms information; ^a^ Including brain NOS; ^b^ p-value by chi2.

**Table A7. Symptoms by laterality**

|  |  | **Laterality N (%)** | | |
| --- | --- | --- | --- | --- |
|  |  | **Contralateral** ^a^ | **Ipsilateral** ^b^ | **No side assigned** ^c^ |
| **Laterality of symptoms** | ***Total no. of symptoms*** | **n (%)** | **n (%)** | **n (%)** |
| **Visual signs & symptoms** | *217* | 15 (7%) | 19 (9%) | 183 (83%) |
| **Focal neurological signs & symptoms** | *287* | 26 (9%) | 20 (7%) | 242 (83%) |
| **Altered sensibility** | *67* | 11 (16%) | 6 (9%) | 50 (75%) |

Percentages by row, based on cases with symptoms information; p- value between contralateral and ipsilateral= 0.33; Cases can have more than one symptom; ^a^ When the symptom occurs on the opposite side of the head where the tumor is located; ^b^ When the symptom occurs on the same side of the head where the tumor is located; ^c^ When cases reported the symptom but no side is assigned and/or the side of the tumor is unknown

**Table A8. Time between occurrence of a symptom and final diagnosis by morphology and topography**

|  |  |  |  |  | **Time between first symptom until final diagnosis** | | | | | |  |  |
| --- | --- | --- | --- | --- | --- | --- | --- | --- | --- | --- | --- | --- |
| **Morphology:** | ***Cases with symptoms (n=722)*** | **n** ^a^ | **months  median  (IQ range)** | **p-value** ^b^ | **0-1 months** | **1-2m** | **2-6m** | **6m-1y** | **1-2 years** | **≥2years** | **NA** | **p-value** ^c^ |
| ***Neuroepithelial:*** | *551* | *490* | *1.40 (0.47-5.17)* |  | *199 (36%)* | *77 (14%)* | *103 (19%)* | *46 (8%)* | *31 (6%)* | *34 (6%)* | *61 (11%)* |  |
| Glioma | 459 | 409 | 1.27 (0.47-4.27) | <0.05 | 177 (39%) | 65 (14%) | 84 (18%) | 37 (8%) | 24 (5%) | 22 (5%) | 50 (11%) | 0.02 |
| Glioma- High grade^e^ | 163 | 152 | 1.10 (0.37-3.02) | <0.05 | 72 (44%) | 29 (18%) | 28 (17%) | 10 (6%) | 8 (5%) | 5 (3%) | 11 (7%) | 0.08 |
| Glioma- Low grade^e^ | 296 | 257 | 1.37 (0.53-5.73) |  | 105 (36%) | 36 (12%) | 56 (19%) | 27 (9%) | 16 (5%) | 17 (6%) | 39 (13%) |  |
| Other neuroepithelial | 92 | 81 | 2.57 (0.70-10.00) |  | 22 (24%) | 12 (13%) | 19 (21%) | 9 (10%) | 7 (7%) | 12 (13%) | 11 (12%) |  |
| ***Non- neuroepithelial:*** | *171* | *148* | *1.45 (0.70-3.88)* |  | *54 (32%)* | *36 (21%)* | *34 (20%)* | *9 (5%)* | *8 (5%)* | *7 (4%)* | *23 (13%)* |  |
| Embryonal | 102 | 93 | 1.47 (0.77-3.57) | 0.67 | 33 (32%) | 25 (24%) | 24 (24%) | 4 (4%) | 4 (4%) | 3 (3%) | 9 (9%) | 0.1 |
| Meningioma | 35 | 26 | 1.22 (0.77-7.83) | 0.98 | 11 (31%) | 6 (17%) | 2 (6%) | 3 (8%) | 1 (3%) | 3 (9%) | 9 (26%) | 0.04 |
| Other non-neuroepithelial | 34 | 29 | 1.70 (0.57-4.87) | 0.59 | 10 (29%) | 5 (15%) | 8 (23%) | 2 (6%) | 3 (9%) | 1 (3%) | 5 (15%) | 0.49 |
| **Topography:** |  |  |  |  |  |  |  |  |  |  |  |  |
| **Brainstem** | 68 | 57 | 1.83 (0.87-4.83) | 0.28 | 16 (24%) | 15 (22%) | 15 (22%) | 6 (9%) | 2 (3%) | 3 (4%) | 11 (16%) | 0.06 |
| **Cerebellum** | 163 | 146 | 1.30 (0.67-3.53) |  | 60 (37%) | 34 (21%) | 27 (17%) | 10 (6%) | 10 (6%) | 5 (3%) | 17 (10%) |  |
| **Frontal lobe** | 113 | 104 | 1.07 (0.33-3.18) |  | 50 (44%) | 21 (19%) | 20 (18%) | 7 (6%) | 2 (2%) | 4 (4%) | 9 (8%) |  |
| **Temporal lobe** | 88 | 80 | 2.02 (0.43-8.02) |  | 31 (35%) | 7 (8%) | 17 (19%) | 11 (13%) | 4 (5%) | 10 (11%) | 8 (9%) |  |
| **Parietal lobe** | 37 | 33 | 2.17 (0.63-3.93) |  | 12 (32%) | 3 (8%) | 13 (35%) | 1 (3%) | 2 (5%) | 2 (5%) | 4 (11%) |  |
| **Occipital lobe** | 16 | 13 | 1.77 (0.67-3.70) |  | 5 (31%) | 2 (13%) | 3 (19%) | 2 (13%) | 1 (6%) | 0 (0%) | 3 (19%) |  |
| **Cerebral ventricles** | 74 | 67 | 1.30 (0.33-7.90) |  | 31 (42%) | 7 (9%) | 11 (15%) | 5 (7%) | 7 (9%) | 6 (8%) | 7 (9%) |  |
| **Cerebral meninges** | 24 | 18 | 1.40 (0.77-2.93) |  | 8 (33%) | 3 (13%) | 3 (13%) | 1 (4%) | 1 (4%) | 2 (8%) | 6 (25%) |  |
| **Cranial nerves** | 21 | 16 | 3.03 (1.67-5.43) |  | 3 (14%) | 1 (5%) | 9 (43%) | 2 (10%) | 1 (5%) | 0 (0%) | 5 (24%) |  |
| **Overlapping lesion** | 76 | 68 | 1.43 (0.53-5.35) |  | 22 (29%) | 14 (18%) | 16 (21%) | 6 (8%) | 5 (7%) | 5 (7%) | 8 (11%) |  |
| **Other parts of brain** ^d^ | 42 | 36 | 1.25 (0.58-9.17) |  | 15 (36%) | 6 (14%) | 3 (7%) | 4 (10%) | 4 (10%) | 4 (10%) | 6 (14%) |  |
| ***Overall*** | *722* | *638* | *1.42 (0.53-4.80)* |  | *253 (35%)* | *113 (16%)* | *137 (19%)* | *55 (8%)* | *39 (5%)* | *41 (6%)* | *84 (12%)* |  |

^a^ number of cases with information on date of first symptom and date of final diagnosis; ^b^ p-value of numeric variables by Kruskal Wallis; ^c^ p-value of categorized variables by chi2; percentage by row (column "Cases with symptoms n=722"); ^d^ Including brain NOS; ^e^High grade (grade I-II) and Low grade (grade III-IV).

**Table A9. Time between EARLIEST and LATEST symptom until final diagnosis in months by age, sex, country, morphology, topography and grade**

|  |  | **Timing of earliest symptoms** | | **Timing of latest symptoms** | |
| --- | --- | --- | --- | --- | --- |
|  | **Cases with information on timing of first or latest symptom (n=638)** | **Median time (months) from earliest symptom to diagnosis** | **p** ^a^ | **Median time (months) from latest symptom to diagnosis** | **p** ^a^ |
|  | **n (%)** | **n (IQR)** |  | **n (IQR)** |  |
| **Age** |  |  | 0.52 |  | 0.78 |
| **10-14** | 269 (42%) | 1.53 (0.60-5.13) |  | 0.70 (0.15-2.22) |  |
| **15-19** | 201 (32%) | 1.33 (0.45-4.38) |  | 0.67 (0.20-2.17) |  |
| **20-24** | 168 (26%) | 1.27 (0.47-4.33) |  | 0.77 (0.23-2.43) |  |
| **Sex** |  |  | 0.56 |  | 0.32 |
| **Male** | 348 (55%) | 1.32 (0.50-4.30) |  | 0.67 (0.20-2.20) |  |
| **Female** | 290 (45%) | 1.47 (0.50-5.00) |  | 0.77 (0.20-2.70) |  |
| **Country** |  |  | <0.05 |  | <0.05 |
| **Australia** | 21 (3%) | 1.53 (0.50-5.37) |  | 1.53 (0.50-2.60) |  |
| **Austria** | 0 (0%) | - |  | - |  |
| **Canada** | 19 (3%) | 3.40 (1.10-24.57) |  | 3.30 (0.77-15.00) |  |
| **France** | 0 (0%) | - |  | - |  |
| **Germany** | 60 (9%) | 2.62 (0.58-6.33) |  | 0.37 (0.00-1.93) |  |
| **Greece** | 47 (8%) | 1.10 (0.50-2.00) |  | 0.70 (0.37-1.67) |  |
| **India** | 21 (3%) | 2.50 (0.73-5.33) |  | 2.20 (0.60-3.63) |  |
| **Israel** | 90 (14%) | 2.20 (0.70-9.47) |  | 0.37 (0.00-1.37) |  |
| **Italy** | 116 (18%) | 1.37 (0.55-4.13) |  | 0.97 (0.28-2.88) |  |
| **N. Zealand** | 12 (2%) | 0.97 (0.63-6.22) |  | 0.67 (0.08-3.17) |  |
| **Spain** | 185 (29%) | 1.07 (0.33-3.07) |  | 0.67 (0.17-2.07) |  |
| **Netherlands** | 15 (2%) | 1.17 (0.67-4.90) |  | 0.67 (0.00-1.40) |  |
| **Japan** | 28 (5%) | 1.78 (1.10-4.22) |  | 1.22 (0.78-2.38) |  |
| **South Korea** | 24 (4%) | 1.08 (0.30-3.40) |  | 0.80 (0.25-1.73) |  |
| **Morphology** |  |  | 0.04 |  | 0.02 |
| **Gliomas** | 409 (64%) | 1.27 (0.47-4.27) |  | 0.63 (0.17-2.20) |  |
| **Other neuroepithelial** | 81 (13%) | 2.52 (0.70-10.00) |  | 1.48 (0.23-4.07) |  |
| **Embryonal** | 93 (15%) | 1.46 (0.77-3.57) |  | 0.70 (0.07-1.33) |  |
| **Meningiomas** | 26 (4%) | 1.22 (0.77-7.83) |  | 0.98 (0.47-9.20) |  |
| **Other non-neuroepithelial** | 29 (4%) | 1.70 (0.57-4.87) |  | 1.17 (0.33-2.83) |  |
| **Topography** |  |  | 0.28 |  | <0.01 |
| **Brainstem** | 57 (9%) | 1.83 (0.87-4.83) |  | 1.00 (0.17-2.43) |  |
| **Cerebellum** | 146 (23%) | 1.30 (0.67-3.53) |  | 0.70 (0.10-1.67) |  |
| **Frontal lobe** | 104 (16%) | 1.07 (0.33-3.18) |  | 0.60 (0.07-1.60) |  |
| **Temporal lobe** | 80 (12%) | 2.02 (0.43-8.02) |  | 1.23 (0.32-5.27) |  |
| **Parietal lobe** | 33 (5%) | 2.17 (0.63-3.93) |  | 1.27 (0.50-3.23) |  |
| **Occipital lobe** | 13 (2%) | 1.77 (0.67-3.70) |  | 0.80 (0.50-3.70) |  |
| **Cerebral ventricle** | 67 (11%) | 1.30 (0.33-7.90) |  | 0.50 (0.10-2.07) |  |
| **Cerebral meninges** | 18 (3%) | 1.40 (0.77-2.93) |  | 0.80 (0.37-3.30) |  |
| **Cranial nerves** | 16 (2%) | 3.03 (1.67-5.43) |  | 2.22 (0.87-4.38) |  |
| **Overlapping** | 68 (11%) | 1.43 (0.53-5.35) |  | 0.40 (0.07-2.37) |  |
| **Other parts of brain** ^b^ | 36 (6%) | 1.25 (0.58-9.17) |  | 0.57 (0.05-1.55) |  |
| **Grade** |  |  | 0.04 |  | 0.03 |
| **I** | 261 (41%) | 1.67 (0.60-7.47) |  | 0.80 (0.23-2.83) |  |
| **II** | 129 (20%) | 1.50 (0.50-5.30) |  | 0.67 (0.17-2.83) |  |
| **III** | 106 (17%) | 1.28 (0.60-3.43) |  | 0.75 (0.17-2.43) |  |
| **IV** | 142 (22%) | 1.22 (0.47-3.00) |  | 0.53 (0.07-1.33) |  |

Percentage by column; empty cell is because Austria and France did not collected information on symptoms; ^a^ p value is chi square calculated by Kruskal-Wallis test comparing the categorical variables (age, sex, country, morphology, topography and grade) vs. timing of earliest or latest symptoms; ^b^ Including brain NOS;

**Table A10. EARLIEST and LATEST symptom(s) before final diagnosis by morphology**

|  | **Earliest** | | | **Latest** | |
| --- | --- | --- | --- | --- | --- |
| **Morphology** | **Cases with symptoms information (n=722)** | **Symptom among earliest (n=638)** | **Months before diagnosis** | **Symptom among latest (n=638)** | **Months before diagnosis** |
|  | **N** | **N** | **median (IQR)** | **N** | **median (IQR)** |
| **Overall** |  | 638 (100%) |  | 638 (100%) |  |
| **Headaches** | 436 | 375 (59%) | 1.27 (0.50-3.40) | 300 (47%) | 0.78 (0.32-2.18) |
| **Nausea/vomiting** | 277 | 212 (33%) | 1.13 (0.48-3.15) | 209 (33%) | 0.70 (0.20-2.00) |
| **Visual signs & symptoms** | 217 | 90 (14%) | 0.80 (0.43-2.27) | 109 (17%) | 0.47 (0.07-1.07) |
| **Focal neurological signs & symptoms** | 288 | 174 (27%) | 1.07 (0.43-3.40) | 202 (32%) | 0.53 (0.13-1.53) |
| **Cognitive, memory and behavioral changes** | 88 | 39 (6%) | 1.53 (0.60-3.67) | 54 (8%) | 0.68 (0.23-2.10) |
| **Convulsions/ seizures** | 174 | 139 (22%) | 1.17 (0.30-5.73) | 145 (23%) | 0.80 (0.23-3.07) |
| **Altered consciousness** | 101 | 68 (11%) | 0.83 (0.27-3.48) | 86 (13%) | 0.50 (0.17-1.53) |
| **Dizziness** | 141 | 94 (15%) | 1.33 (0.57-3.40) | 95 (15%) | 1.10 (0.37-3.23) |
| **Altered sensibility** | 67 | 33 (5%) | 2.67 (0.70-5.30) | 36 (6%) | 0.73 (0.05-3.90) |
| Neuroepithelial: |  |  |  |  |  |
| Glioma |  | 459 (100%) |  | 459 (100%) |  |
| **Headaches** | 280 | 239 (52%) | 1.03 (0.47-3.23) | 202 (44%) | 0.73 (0.27-2.30) |
| **Nausea/vomiting** | 178 | 135 (29%) | 1.07 (0.53-3.67) | 137 (30%) | 0.70 (0.20-2.17) |
| **Visual signs & symptoms** | 133 | 55 (12%) | 0.70 (0.33-2.50) | 70 (15%) | 0.40 (0.03-0.77) |
| **Focal neurological signs & symptoms** | 185 | 109 (24%) | 0.90 (0.27-2.67) | 122 (27%) | 0.47 (0.10-1.17) |
| **Cognitive, memory and behavioral changes** | 49 | 25 (5%) | 1.53 (0.70-3.67) | 34 (7%) | 0.75 (0.40-2.30) |
| **Convulsions/seizures** | 106 | 86 (19%) | 0.87 (0.27-3.93) | 92 (20%) | 0.63 (0.17-3.30) |
| **Altered consciousness** | 64 | 50 (11%) | 0.65 (0.17-3.67) | 56 (12%) | 0.40 (0.10-1.13) |
| **Dizziness** | 88 | 54 (12%) | 1.40 (0.47-3.53) | 59 (13%) | 1.07 (0.20-3.40) |
| **Altered sensibility** | 42 | 19 (4%) | 2.67 (0.77-5.07) | 23 (5%) | 0.67 (0.03-3.67) |
| Other neuroepithelial |  | 92 (100%) |  | 92 (100%) |  |
| **Headaches** | 42 | 33 (36%) | 1.53 (0.30-4.83) | 22 (24%) | 0.73 (0.23-2.07) |
| **Nausea/vomiting** | 17 | 14 (15%) | 0.78 (0.23-4.90) | 11 (12%) | 0.23 (0.20-0.87) |
| **Visual signs & symptoms** | 19 | 5 (5%) | 1.07 (0.70-1.27) | 6 (7%) | 0.75 (0.30-1.27) |
| **Focal neurological signs & symptoms** | 23 | 13 (14%) | 3.63 (2.30-7.53) | 16 (17%) | 0.98 (0.20-4.43) |
| **Cognitive, memory and behavioral changes** | 15 | 4 (4%) | 1.77 (0.52-5.22) | 9 (10%) | 0.60 (0.33-2.93) |
| **Convulsions/seizures** | 49 | 39 (42%) | 2.33 (0.40-10.00) | 37 (40%) | 1.50 (0.23-3.20) |
| **Altered consciousness** | 22 | 10 (11%) | 2.17 (1.27-4.33) | 17 (18%) | 2.00 (1.07-3.80) |
| **Dizziness** | 9 | 8 (9%) | 3.10 (0.93-9.85) | 7 (8%) | 1.97 (0.43-4.23) |
| **Altered sensibility** | 9 | 5 (5%) | 5.30 (2.93-7.50) | 6 (7%) | 3.15 (0.00-5.60) |
| Non- neuroepithelials |  |  |  |  |  |
| Embryonal |  | 102 (100%) |  | 102 (100%) |  |
| **Headaches** | 79 | 74 (73%) | 1.33 (0.77-3.07) | 53 (52%) | 1.10 (0.50-1.67) |
| **Nausea/vomiting** | 69 | 54 (53%) | 1.28 (0.67-2.50) | 51 (50%) | 0.93 (0.27-1.67) |
| **Visual signs & symptoms** | 39 | 16 (16%) | 1.08 (0.73-1.42) | 22 (22%) | 0.63 (0.07-1.10) |
| **Focal neurological signs & symptoms** | 53 | 33 (32%) | 1.10 (0.50-3.07) | 46 (45%) | 0.63 (0.07-1.53) |
| **Cognitive, memory and behavioral changes** | 17 | 6 (6%) | 1.78 (0.37-3.57) | 6 (6%) | 0.23 (0.07-0.50) |
| **Convulsions/seizures** | 6 | 3 (3%) | 5.33 (0.00-117.33) | 3 (3%) | 0.37 (0.00-0.87) |
| **Altered consciousness** | 10 | 5 (5%) | 0.37 (0.33-2.50) | 8 (8%) | 0.35 (0.00-0.52) |
| **Dizziness** | 32 | 24 (24%) | 1.13 (0.70-1.90) | 22 (22%) | 0.95 (0.67-1.67) |
| **Altered sensibility** | 6 | 5 (5%) | 0.37 (0.33-0.70) | 4 (4%) | 0.35 (0.17-0.53) |
| Meningioma |  | 35 (100%) |  | 35 (100%) |  |
| **Headaches** | 13 | 9 (26%) | 1.43 (0.77-2.93) | 8 (23%) | 1.25 (0.63-5.43) |
| **Nausea/vomiting** | 3 | 3 (9%) | 1.43 (1.07-1.67) | 3 (9%) | 1.43 (1.07-1.67) |
| **Visual signs & symptoms** | 17 | 8 (23%) | 2.08 (0.72-5.38) | 5 (14%) | 0.63 (0.47-0.80) |
| **Focal neurological signs & symptoms** | 13 | 10 (29%) | 0.90 (0.77-17.33) | 8 (23%) | 0.90 (0.62-13.58) |
| **Cognitive, memory and behavioral changes** | 2 | 2 (6%) | 13.92 (0.80-27.03) | 2 (6%) | 13.92 (0.80-27.03) |
| **Convulsions/seizures** | 11 | 10 (29%) | 0.78 (0.27-1.00) | 11 (31%) | 0.80 (0.27-1.37) |
| **Altered consciousness** | 2 | 2 (6%) | 0.62 (0.47-0.77) | 2 (6%) | 0.62 (0.47-0.77) |
| **Dizziness** | 4 | 3 (9%) | 1.43 (0.80-9.20) | 3 (9%) | 1.43 (0.80-9.20) |
| **Altered sensibility** | 3 | 0 | - | - | - |
| Other non-neuroepithelial |  | 34 (100%) |  | 34 (100%) |  |
| **Headaches** | 22 | 20 (59%) | 1.93 (0.47-5.90) | 15 (44%) | 1.67 (0.33-2.87) |
| **Nausea/vomiting** | 10 | 6 (18%) | 0.50 (0.00-1.30) | 7 (21%) | 0.33 (0.00-1.70) |
| **Visual signs & symptoms** | 9 | 6 (18%) | 0.57 (0.33-6.30) | 6 (18%) | 0.55 (0.33-0.63) |
| **Focal neurological signs & symptoms** | 13 | 9 (26%) | 2.87 (0.57-6.30) | 10 (29%) | 0.62 (0.27-2.87) |
| **Cognitive, memory and behavioral changes** | 5 | 2 (6%) | 0.43 (0.20-0.67) | 3 (9%) | 0.23 (0.20-0.67) |
| **Convulsions/seizures** | 2 | 1 (3%) | 1.17 (1.17-1.17) | 2 (6%) | 0.88 (0.60-1.17) |
| **Altered consciousness** | 3 | 1 (3%) | 0.50 (0.50-0.50) | 3 (9%) | 0.50 (0.23-1.17) |
| **Dizziness** | 8 | 5 (15%) | 0.50 (0.43-2.87) | 4 (12%) | 0.47 (0.22-1.68) |
| **Altered sensibility** | 7 | 4 (12%) | 6.43 (3.52-10.65) | 3 (9%) | 4.87 (2.17-8.00) |

Percentage by morphology type; empty cell is because any participant had this characteristic

**Table A11. Median time lag (months) of earliest and latest symptoms and diagnosis by age, morphology and topography**

|  | **10-14** | | | **15-19** | | | **20-24** | | |
| --- | --- | --- | --- | --- | --- | --- | --- | --- | --- |
|  | **EARLIEST** | | **LATEST** | **EARLIEST** | | **LATEST** | **EARLIEST** | | **LATEST** |
| **MORPHOLOGIES** | **n** | **median (IQR)** | **median (IQR)** | **n** | **median (IQR)** | **median (IQR)** | **n** | **median (IQR)** | **median (IQR)** |
| **Diffuse astrocytic & oligodendrocytic tumors** | 44 | 1.05 (0.38-2.58) | 0.57 (0.22-1.87) | 43 | 0.90 (0.33-5.17) | 0.43 (0.10-2.63) | 71 | 1.00 (0.40-3.40) | 0.77 (0.17-2.03) |
| **Other astrocytic tumors** | 82 | 1.40 (0.53-5.80) | 0.62 (0.07-2.27) | 56 | 2.02 (0.72-5.63) | 0.77 (0.20-2.52) | 24 | 2.38 (0.88-7.80) | 0.45 (0.23-1.73) |
| **Ependymal tumors** | 17 | 1.27 (0.73-3.00) | 0.80 (0.60-1.73) | 15 | 0.60 (0.13-2.50) | 0.23 (0.00-0.63) | 10 | 1.30 (0.07-4.27) | 0.18 (0.07-2.43) |
| **Other gliomas** | 30 | 1.80 (0.57-12.43) | 0.90 (0.17-6.33) | 12 | 1.93 (0.78-5.00) | 0.47 (0.25-1.93) | 8 | 1.13 (0.45-3.20) | 0.83 (0.20-3.20) |
| **Choroid plexus tumors** | 4 | 0.57 (0.42-0.97) | 0.42 (0.30-0.57) | 1 | 24.97 (24.97-24.97) | 0.60 (0.60-0.60) | 5 | 4.50 (0.30-5.50) | 2.43 (0.30-4.50) |
| **Neuronal & mixed neuronal-glial tumors** | 31 | 4.90 (1.27-9.20) | 1.43 (0.20-6.53) | 32 | 1.28 (0.30-3.28) | 0.97 (0.23-2.00) | 15 | 5.40 (2.30-24.73) | 3.07 (1.53-24.73) |
| **Embryonal tumors** | 57 | 1.57 (0.80-3.63) | 0.70 (0.00-1.53) | 24 | 1.28 (0.75-3.10) | 0.75 (0.28-1.40) | 12 | 0.85 (0.48-2.18) | 0.50 (0.22-1.05) |
| **Tumors of cranial nerves** | 2 | 8.60 (3.90-13.30) | 3.02 (2.13-3.90) | 4 | 3.52 (1.67-5.58) | 1.67 (0.67-3.52) | 5 | 2.87 (0.57-3.20) | 1.17 (0.57-2.87) |
| **Meningiomas** | 0 | - | - | 13 | 1.67 (0.80-9.20) | 1.43 (0.63-9.20) | 13 | 0.97 (0.63-1.50) | 0.80 (0.27-1.37) |
| **Other mesenchymal tumors** | 2 | 7.18 (1.67-12.70) | 7.18 (1.67-12.70) | 1 | 1.23 (1.23-1.23) | 0.23 (0.23-0.23) | 5 | 0.67 (0.00-1.70) | 0.67 (0.00-1.70) |
| **TOPOGRAPHIES** | **10-14** | | | **15-19** | | | **20-24** | | |
|  | **EARLIEST** | | **LATEST** | **EARLIEST** | | **LATEST** | **EARLIEST** | | **LATEST** |
|  | **n** | **median (IQR)** | **median (IQR)** | **n** | **median (IQR)** | **median (IQR)** | **n** | **median (IQR)** | **median (IQR)** |
| **Brainstem** | 35 | 1.40 (0.57-3.20) | 1.03 (0.17-2.30) | 16 | 1.82 (0.97-4.38) | 0.33 (0.12-1.82) | 6 | 11.25 (4.43-30.40) | 3.55 (0.37-10.53) |
| **Cerebellum** | 80 | 1.53 (0.70-4.60) | 0.70 (0.07-1.67) | 37 | 1.27 (0.77-2.57) | 0.77 (0.33-1.47) | 29 | 0.87 (0.27-2.60) | 0.53 (0.17-1.47) |
| **Frontal lobe** | 25 | 1.73 (0.33-3.93) | 0.53 (0.00-1.73) | 36 | 0.63 (0.30-1.45) | 0.43 (0.07-1.17) | 43 | 1.10 (0.47-3.30) | 0.83 (0.27-2.43) |
| **Temporal lobe** | 23 | 3.20 (0.53-7.53) | 1.70 (0.33-6.30) | 33 | 1.50 (0.40-12.60) | 0.93 (0.40-3.63) | 24 | 2.02 (0.68-6.37) | 1.27 (0.23-4.10) |
| **Parietal lobe** | 14 | 3.07 (1.27-4.23) | 2.25 (0.50-4.07) | 11 | 2.17 (0.63-5.73) | 1.07 (0.63-3.23) | 8 | 0.53 (0.17-1.75) | 0.53 (0.17-1.38) |
| **Occipital lobe** | 5 | 1.07 (0.80-7.30) | 1.07 (0.80-6.30) | 6 | 1.22 (0.50-2.23) | 0.70 (0.50-2.23) | 2 | 6.03 (3.33-8.73) | 4.52 (0.30-8.73) |
| **Cerebral ventricles** | 28 | 0.97 (0.62-5.45) | 0.63 (0.13-1.37) | 23 | 2.50 (0.23-9.20) | 0.27 (0.00-1.97) | 16 | 1.18 (0.35-6.00) | 0.40 (0.18-2.90) |
| **Cerebral meninges** | 0 | - | - | 9 | 1.43 (0.77-7.83) | 0.80 (0.47-3.30) | 9 | 1.37 (0.80-2.30) | 0.80 (0.30-1.37) |
| **Cranial nerves** | 6 | 3.08 (0.23-5.80) | 2.20 (0.23-3.90) | 5 | 2.73 (2.17-4.87) | 2.17 (1.17-2.73) | 5 | 3.20 (2.87-5.07) | 2.87 (1.17-5.07) |
| **Overlapping lesion** | 39 | 2.17 (0.73-6.40) | 0.40 (0.03-2.17) | 10 | 1.08 (0.33-2.57) | 0.50 (0.13-2.57) | 19 | 1.00 (0.40-5.40) | 0.40 (0.20-2.83) |
| **Other parts of brain** ^a^ | 14 | 0.82 (0.17-2.10) | 0.40 (0.00-0.77) | 15 | 6.27 (0.47-13.13) | 0.60 (0.00-12.17) | 7 | 1.27 (0.87-3.43) | 0.77 (0.10-1.27) |

^a^ Including brain NOS; empty cell is because any participant had this characteristic

**Table A12. Sequence of symptoms by morphology and topography**

|  |  |  |  |  | **Morphology** | | | | | **Topography** | | | | | | | | | | | **Total** |
| --- | --- | --- | --- | --- | --- | --- | --- | --- | --- | --- | --- | --- | --- | --- | --- | --- | --- | --- | --- | --- | --- |
|  |  |  |  | **Gliomas** |  | **Other neuroep. (n= 92)** | **Embryonal tumors (n=102)** | **Meningiomas (n=35)** | **Other non neuroepithelial (n=34)** | **Brainstem (n=68)** | **Cerebellum (n=163)** | **Frontal lobe (n=113)** | **Temporal lobe (n=88)** | **Parietal lobe (n=37)** | **Occipital lobe (n=16)** | **Cerebral ventricles (n=74)** | **Cerebral meninges (n=24)** | **Cranial nerves (n=21)** | **Overlapping (n=76)** | **Other parts of brain**^a^ **(n=42)** |  |
|  |  |  | **All gliomas (n=459)** | **Glioma- High grade (n=163)^b^** | **Glioma- Low grade (n=296)^b^** |  |  |  |  |  |  |  |  |  |  |  |  |  |  |  |  |
| **Headaches as first symptom** | |  | 238 (52%) | 92 (56%) | 146 (49%) | 33 (36%) | 74 (73%) | 9 (26%) | 20 (59%) | 37 (54%) | 119 (73%) | 40 (35%) | 31 (35%) | 15 (41%) | 5 (31%) | 51 (19%) | 7 (29%) | 8 (38%) | 36 (47%) | 25 (60%) | 374 |
|  | **Nausea/vomiting as second symptom** | | 131 (29%) | 57 (35%) | 74 (25%) | 11 (12%) | 55 (54%) | 3 (9%) | 8 (24%) | 24 (35%) | 83 (51%) | 18 (16%) | 12 (14%) | 7 (19%) | 2 (13%) | 27 (10%) | 1 (4%) | 2 (10%) | 17 (22%) | 15 (36%) | 208 |
|  | **Focal neurological sign and symptom** | | 48 (10%) | 18 (11%) | 30 (10%) | 2 (2%) | 25 (25%) | 1 (3%) | 6 (18%) | 16 (24%) | 36 (22%) | 3 (3%) | 4 (5%) | 1 (3%) | - | 6 (2%) | 1 (4%) | 2 (10%) | 9 (12%) | 4 (10%) | 82 |
| **Nausea/vomiting as first symptom** | | | 30 (7%) | 10 (6%) | 20 (7%) | 5 (5%) | 8 (8%) | - | 1 (3%) | 7 (10%) | 11 (7%) | 10 (9%) | 3 (3%) | 2 (5%) | - | 5 (2%) | - | - | 5 (7%) | 1 (2%) | 44 |
|  | **Focal neurological sign and symptoms** | | 10 (2%) | 3 (2%) | 7 (2%) | 1 (1%) | 3 (3%) | - | 1 (3%) | 5 (7%) | 4 (2%) | 2 (2%) | - | - | - | 1 (0%) | - | - | 3 (4%) | - | 15 |
|  | **Headaches** | | 7 (2%) | 4 (2%) | 3 (1%) | - | 1 (1%) | - | 1 (3%) | 3 (4%) | 2 (1%) | 1 (1%) | - | 1 (3%) | - | 1 (0%) | - | - | 1 (1%) | - | 9 |
| **Visual sign and symptoms** | |  | 23 (5%) | 7 (4%) | 16 (5%) | 3 (3%) | 3 (3%) | 5 (14%) | 3 (9%) | 3 (4%) | 5 (3%) | - | 4 (5%) | 1 (3%) | 3 (19%) | 3 (1%) | 5 (21%) | 3 (14%) | 7 (9%) | 3 (7%) | 37 |
|  | **Headaches as second symptom** | | 6 (1%) | 1 (1%) | 5 (2%) | - | 1 (1%) | 2 (6%) | 1 (3%) | 1 (1%) | 2 (1%) | - | - | - | 1 (6%) | 1 (0%) | 2 (8%) | - | 3 (4%) | - | 10 |
|  | **Nausea/vomiting as third symptom** | | 3 (1%) | 0 (0%) | 3 (1%) | - | 1 (1%) | - | - | - | 2 (1%) | - | - | - | - | - | - | - | 1 (1%) | - | 3 |
| **Focal neurological sign and symptoms** | | | 41 (9%) | 16 (10%) | 25 (8%) | 6 (7%) | 4 (4%) | 6 (17%) | 2 (6%) | 10 (15%) | 7 (4%) | 11 (10%) | 8 (9%) | 5 (14%) | 1 (6%) | 2 (1%) | 1 (4%) | 2 (10%) | 9 (12%) | 3 (7%) | 59 |
|  | **Headaches as second symptom** | | 14 (3%) | 5 (3%) | 9 (3%) | - | 2 (2%) | - | 2 (6%) | 6 (9%) | 3 (2%) | 2 (2%) | - | 1 (3%) | - | - | - | 2 (10%) | 1 (1%) | 3 (7%) | 18 |
|  | **Nausea/vomiting as third symptom** | | 10 (2%) | 4 (%) | 6 (2%) | 1 (1%) | 1 (1%) | - | 1 (3%) | 4 (6%) | 1 (1%) | 2 (2%) | - | 1 (3%) | - | 1 (0%) | - | 1 (5%) | 2 (3%) | 1 (2%) | 13 |
| **Convulsions/seizures** | |  | 59 (13%) | 21 (13%) | 38 (13%) | 27 (29%) | 1 (1%) | 6 (17%) | 1 (3%) | - | 2 (1%) | 37 (33%) | 29 (33%) | 8 (22%) | 3 (19%) | 2 (1%) | 5 (21%) | 1 (5%) | 6 (8%) | 1 (2%) | 94 |
|  | **Altered consciousness** | | 12 (3%) | 3 (2%) | 9 (3%) | 3 (3%) | - | - | - | - | - | 7 (6%) | 5 (6%) | 1 (3%) | - | 1 (0%) | - | - | 1 (1%) | - | 15 |
|  | **Headaches** | | 2 (0%) | 1 (1%) | 1 (0%) | 1 (1%) | 1 (1%) | - | - | - | - | 1 (1%) | 1 (1%) | - | - | - | - | - | 1 (1%) | - | 3 |

Percentages by column, based on cases with symptoms information (n=722); ^a^ Including brain NOS; empty cell is because any participant had this characteristic; ^b^High grade (grade I-II) and Low grade (grade III-IV).

**Table A13. Cases with rare morphologies (n=5-30)**

| **ICD code** | **Morphology** | **No.** | **Gender** | | **Age (years)** | | | **Topography** | |
| --- | --- | --- | --- | --- | --- | --- | --- | --- | --- |
|  |  | **n** | **M** | **F** | **10-14** | **15-19** | **20-24** | **Type** | **N** |
| 9539/1 | Atypical meningioma | 8 | 4 | 4 | - | 3 | 5 | Frontal lobe | 1 |
|  |  |  |  |  |  |  |  | Temporal lobe | 2 |
|  |  |  |  |  |  |  |  | Cerebral meninges | 5 |
| 9506/1 | Central neurocytoma | 18 | 13 | 5 | 4 | 8 | 6 | Frontal lobe | 1 |
|  |  |  |  |  |  |  |  | Temporal lobe | 1 |
|  |  |  |  |  |  |  |  | Cerebral ventricles | 13 |
|  |  |  |  |  |  |  |  | Other parts of brain | 3 |
| 9390/0 | Choroid plexus papilloma, NOS | 14 | 10 | 4 | 4 | 4 | 6 | Other parts of brain | 14 |
| 9412/1 | Desmoplastic infantile astrocytoma | 5 | 3 | 2 | 3 | 2 | - | Frontal lobe | 2 |
|  |  |  |  |  |  |  |  | Temporal lobe | 3 |
| 9471/3 | Desmoplastic nodular medulloblastoma | 25 | 17 | 8 | 11 | 9 | 5 | Brainstem | 2 |
|  |  |  |  |  |  |  |  | Cerebellum | 20 |
|  |  |  |  |  |  |  |  | Other parts of brain | 3 |
| 9413/0 | Dysembryoplastic neuroepithelial tumor | 23 | 13 | 10 | 14 | 6 | 3 | Frontal lobe | 2 |
|  |  |  |  |  |  |  |  | Temporal lobe | 11 |
|  |  |  |  |  |  |  |  | Other parts of brain | 10 |
| 9392/3 | Ependymoma, anaplastic | 21 | 9 | 12 | 7 | 8 | 6 | Brainstem | 4 |
|  |  |  |  |  |  |  |  | Cerebellum | 5 |
|  |  |  |  |  |  |  |  | Frontal lobe | 7 |
|  |  |  |  |  |  |  |  | Other parts of brain | 5 |
| 9532/0 | Fibrous meningioma | 5 | 2 | 3 | 1 | 3 | 1 | Other parts of brain | 5 |
| 9451/3 | Glioma, malignant | 1 | - | 1 | 1 | - | - | Parietal lobe | 1 |
|  | Oligodendroglioma, anaplastic | 7 | 4 | 3 | 1 | 3 | 3 | Frontal lobe | 1 |
|  |  |  |  |  |  |  |  | Temporal lobe | 1 |
|  |  |  |  |  |  |  |  | Other parts of brain | 5 |
| 9161/1 | Hemangioblastoma | 10 | 5 | 5 | 1 | 3 | 6 | Cerebellum | 7 |
|  |  |  |  |  |  |  |  | Other parts of brain | 3 |
| 9474/3 | Large cell medulloblastoma | 9 | 8 | 1 | 5 | 3 | 1 | Brainstem | 1 |
|  |  |  |  |  |  |  |  | Cerebellum | 7 |
|  |  |  |  |  |  |  |  | Other parts of brain | 1 |
| 9530/0 | Meningioma, NOS | 11 | 5 | 6 | - | 3 | 8 | Cerebellum | 1 |
|  |  |  |  |  |  |  |  | Frontal lobe | 1 |
|  |  |  |  |  |  |  |  | Temporal lobe | 1 |
|  |  |  |  |  |  |  |  | Other parts of brain | 8 |
| 9531/0 | Meningothelial meningioma | 10 | 6 | 4 | 1 | 5 | 4 | Cerebral meninges | 9 |
|  |  |  |  |  |  |  |  | Other parts of brain | 1 |
| 9382/3 | Mixed glioma | 27 | 20 | 7 | 3 | 7 | 17 | Frontal lobe | 16 |
|  |  |  |  |  |  |  |  | Temporal lobe | 4 |
|  |  |  |  |  |  |  |  | Other parts of brain | 7 |
| 9560/0 | Neurilemmoma, NOS | 14 | 7 | 7 | 1 | 4 | 9 | Brainstem | 1 |
|  |  |  |  |  |  |  |  | Cerebellum | 1 |
|  |  |  |  |  |  |  |  | Other parts of brain | 12 |
| 9560/1 | Neurinomatosis | 5 | 4 | 1 | 3 | 2 | 0 | Temporal lobe | 1 |
|  |  |  |  |  |  |  |  | Other parts of brain | 4 |
| 9451/3 | Oligodendroglioma, anaplastic | 7 | 4 | 3 | 3 | 5 | 11 | Frontal lobe | 10 |
|  |  |  |  |  |  |  |  | Temporal lobe | 4 |
|  |  |  |  |  |  |  |  | Other parts of brain | 5 |
| 9450/3 | Oligodendroglioma, NOS | 19 | 11 | 8 | 1 | 3 | 3 | Frontal lobe | 1 |
|  |  |  |  |  |  |  |  | Temporal lobe | 1 |
|  |  |  |  |  |  |  |  | Other parts of brain | 5 |
| 9509/1 | Papillary glioneuronal tumor | 13 | 7 | 6 | 3 | 10 | - | Brainstem | 1 |
|  |  |  |  |  |  |  |  | Cerebellum | 2 |
|  |  |  |  |  |  |  |  | Frontal lobe | 1 |
|  |  |  |  |  |  |  |  | Temporal lobe | 3 |
|  |  |  |  |  |  |  |  | Other parts of brain | 6 |
| 9424/3 | Pleomorphic xanthoastrocytoma | 16 | 7 | 9 | 7 | 5 | 4 | Cerebellum | 2 |
|  |  |  |  |  |  |  |  | Frontal lobe | 2 |
|  |  |  |  |  |  |  |  | Temporal lobe | 4 |
|  |  |  |  |  |  |  |  | Other parts of brain | 8 |
| 9473/3 | Primitive neuroectodermal tumor, NOS | 10 | 7 | 3 | 4 | 6 | - | Frontal lobe | 2 |
|  |  |  |  |  |  |  |  | Temporal lobe | 2 |
|  |  |  |  |  |  |  |  | Other parts of brain | 6 |
| 9537/0 | Transitional meningioma | 9 | 3 | 6 | 2 | 5 | 2 | Frontal lobe | 3 |
|  |  |  |  |  |  |  |  | Other parts of brain | 6 |
| Overall |  | 287 | 169 | 118 | 80 | 107 | 100 |  |  |

Empty cell is because any participant had this characteristic

Abbreviations:M=male; F=female

**Table A14. Time (in months) between symptom onset and final diagnosis by rare morphologies (n=5-30)**

| **ICD code** | **Morphology** | **Time symptoms until diagnosis (months)** | | | | | | | | |
| --- | --- | --- | --- | --- | --- | --- | --- | --- | --- | --- |
|  |  | **Headaches** | **Nausea/vomiting** | **Visual signs & symptoms** | **Focal neurological signs & symptoms** | **Cognitive, memory and behavioral change** | **Convulsions/seizures** | **Altered consciousness** | **Dizziness** | **Altered sensibility** |
|  |  | **number of cases  (min - max lag time)** | **number of cases  (min - max lag time)** | **number of cases  (min - max lag time)** | **number of cases  (min - max lag time)** | **number of cases  (min - max lag time)** | **number of cases  (min - max lag time)** | **number of cases  (min - max lag time)** | **number of cases  (min - max lag time)** | **number of cases  (min - max lag time)** |
| 9539/1 | Atypical meningioma | 2 (0.77-2.93) | - | 2 (0.00-2.93) | 3 (0.77-27.03) | 1 (27.03-27.03) | 2 (0.80-9.83) | - | - | - |
| 9506/1 | Central neurocytoma | 10 (0.00-72.67) | 4 (0.20-5.40) | 3 (0.30-0.70) | 4 (0.30-5.30) | 1 (0.20-0.20) | 4 (0.00-2.83) | 3 (0.00-0.30) | 1 (1.97-1.97) | 5 (0.00-74.70) |
| 9390/0 | Choroid plexus papilloma NOS | 6 (0.30-24.97) | 2 (1.30-2.43) | 4 (0.20-0.63) | 3 (0.27-2.63) | 1 (0.20-0.20) | 1 (0.60-0.60) | 1 (0.50-0.50) | 2 (0.50-3.67) | - |
| 9412/1 | Desmoplastic infantile astrocytoma | 1 (3.87-3.87) | 2 (4.90-8.10) | - | 1 (0.00-0.00) | - | 2 (8.10-9.20) | - | - | - |
| 9471/3 | Desmoplastic nodular medulloblastoma | 16 (0.00-6.27) | 14 (0.00-3.53) | 5 (0.47-6.27) | 10 (0.00-6.27) | - | - | - | 6 (0.70-5.00) | 1 (0.70-0.70) |
| 9413/0 | Dysembryoplastic neuroepithelial tumor | 5 (0.03-36.93) | 3 (0.37-82.57) | 1 (1.27-1.27) | 3 (0.33-82.57) | 1 (0.33-0.33) | 11 (0.00-82.57) | 6 (0.17-39.93) | 1 (0.23-0.23) | - |
| 9392/3 | Ependymoma anaplastic | 10 (0.00-13.43) | 10 (0.60-4.27) | 4 (0.17-4.27) | 6 (0.60-3.07) | - | 1 (2.43-2.43) | 1 (0.17-0.17) | 4 (0.00-6.20) | 2 (0.67-3.07) |
| 9532/0 | Fibrous meningioma | - | - | 1 (0.80-0.80) | 1 (0.80-0.80) | 1 (0.80-0.80) | 1 (0.37-0.37) | - | 1 (0.80-0.80) | - |
| 9161/1 | Hemangioblastoma | 7 (0.00-2.83) | 5 (0.00-1.70) | - | 3 (0.00-0.67) | 2 (0.23-0.67) | - | 1 (0.23-0.23) | 1 (0.00-0.00) | - |
| 9474/3 | Large cell medulloblastoma | 6 (0.83-25.20) | 7 (0.33-25.20) | 1 (0.00-0.00) | 5 (0.00-1.47) | - | - | 3 (0.33-2.50) | 1 (0.80-0.80) | 1 (0.33-0.33) |
| 9530/0 | Meningioma NOS | 3 (0.63-9.20) | 1 (1.67-1.67) | 1 (0.63-0.63) | 3 (0.00-1.00) | - | 4 (0.00-1.00) | 1 (0.77-0.77) | 1 (9.20-9.20) | - |
| 9531/0 | Meningothelial meningioma | 1 (1.43-1.43) | 1 (1.43-1.43) | 1 (7.83-7.83) | 1 (101.70-101.70) | - | 2 (0.00-3.30) | - | 1 (1.43-1.43) | - |
| 9382/3 | Mixed glioma | 3 (0.03-0.60) | - | 3 (0.03-0.77) | 3 (0.03-0.77) | - | 12 (0.07-12.60) | 3 (0.07-1.10) | 1 (3.40-3.40) | - |
| 9560/0 | Neurilemmoma NOS | 4 (0.43-6.30) | - | 1 (6.30-6.30) | 3 (0.57-6.30) | - | - | - | 2 (0.43-2.87) | 2 (2.17-4.87) |
| 9560/1 | Neurinomatosis | 1 (13.30-13.30) | - | 1 (8.00-8.00) | 3 (3.90-13.30) | - | - | - | - | 2 (8.00-13.30) |
| 9451/3 | Oligodendroglioma anaplastic | 2 (0.60-0.73) | 1 (0.73-0.73) | - | 2 (1.30-10.63) | 2 (0.70-13.63) | 10 (0.07-76.33) | 3 (0.40-10.63) | 1 (10.63-10.63) | 1 (1.30-1.30) |
| 9450/3 | Oligodendroglioma NOS | 5 (0.33-3.40) | 4 (0.17-3.23) | - | - | - | 1 (4.80-4.80) | 1 (0.17-0.17) | 3 (0.87-3.40) | - |
| 9509/1 | Papillary glioneuronal tumor | 5 (0.00-62.87) | 2 (0.23-0.87) | 2 (1.07-3.80) | 2 (2.93-12.97) | 2 (2.93-3.80) | 3 (0.40-27.90) | 4 (0.50-4.33) | 1 (12.97-12.97) | 1 (2.93-2.93) |
| 9424/3 | Pleomorphic xanthoastrocytoma | 7 (0.10-8.73) | 3 (0.10-2.17) | 4 (0.10-7.93) | 4 (0.67-4.60) | 1 (0.50-0.50) | 7 (0.00-3.93) | 4 (0.03-8.73) | - | 2 (0.00-1.13) |
| 9473/3 | Primitive neuroectodermal tumor NOS | 5 (0.50-5.33) | 4 (0.07-3.30) | - | 4 (0.00-3.30) | 2 (0.00-2.03) | 2 (0.00-5.33) | 2 (0.00-0.00) | 1 (1.10-1.10) | - |
| 9537/0 | Transitional meningioma | 3 (0.47-17.33) | 1 (1.07-1.07) | 1 (0.47-0.47) | 2 (0.47-17.33) | - | 1 (1.37-1.37) | 1 (0.47-0.47) | - | - |

Empty cell is because any participant had this characteristic

**Table A15. Time between first image until final diagnosis by morphology**

|  |  |  |  |  | **Time between first image until final diagnosis** | | | |  |  |  |  |
| --- | --- | --- | --- | --- | --- | --- | --- | --- | --- | --- | --- | --- |
| **Morphology:** | ***Number total of cases*** | **n** ^a^ | **months  median  (IQ range)** | **p-value** ^b^ | **0-1 months (n(%))** | **1-2 months (n(%))** | **2-6 months (n(%))** | **6m to <1 year  (n(%))** | **1 to 2 years  (n(%))** | **> 2 years  (n(%))** | **NA** | **p-value** ^c^ |
| ***Neuroepithelial:*** | *676* | *671* | *0.27 (0.10-0.87)* |  | *514 (76%)* | *65 (10%)* | *68 (10%)* | *12 (2%)* | *7 (1%)* | *2 (0%)* | *8 (1%)* |  |
| Glioma | 556 | 552 | 0.23 (0.10-0.77) | <0.05 | 438 (79%) | 50 (9%) | 48 (9%) | 8 (1%) | 4 (1%) | 1 (0%) | 7 (1%) | <0.05 |
| Glioma- High grade^d^ | 201 | 200 | 0.20 (0.07-0.63) |  | 163 (81%) | 18 (9%) | 14 (7%) | 2 (1%) | 1 (1%) | 0 (0%) | 3 (2%) |  |
| Glioma- Low grade^d^ | 355 | 352 | 0.27 (0.10-0.82) | 0.07 | 275 (78%) | 32 (9%) | 34 (10%) | 6 (2%) | 3 (1%) | 1 (0%) | 4 (1%) | 0.90 |
| Other neuroepithelial | 120 | 119 | 0.47 (0.13-1.90) |  | 76 (63%) | 15 (12%) | 20 (17%) | 4 (3%) | 3 (3%) | 1 (1%) | 1 (1%) |  |
| ***Non neuroepithelial:*** | *223* | *218* | *0.23 (0.10-0.70)* |  | *171 (77%)* | *24 (11%)* | *14 (7%)* | *3 (1%)* | *5 (2%)* | *1 (0%)* | *5 (2%)* |  |
| Embryonal | 129 | 126 | 0.17 (0.07-0.33) | <0.05 | 112 (87%) | 7 (5%) | 3 (2%) | 1 (1%) | 1 (1%) | 1 (1%) | 4 (3%) | <0.05 |
| Meningioma | 47 | 47 | 0.60 (0.23-1.83) | <0.05 | 27 (57%) | 10 (21%) | 4 (9%) | 2 (4%) | 4 (9%) | - | - | <0.05 |
| Other non-neuroepithelial | 47 | 46 | 0.35 (0.13-1.07) | 0.13 | 32 (68%) | 7 (15%) | 7 (15%) | - | - | - | 1 (2%) | 0.05 |
| **Overall** | 899 | 890 | 0.27 (0.10-0.83) | <0.05 | 685 (76%) | 89 (10%) | 82 (9%) | 15 (2%) | 12 (1%) | 3 (0%) | 13 (2%) | <0.05 |

^a^ Number of cases with information of date of first image and date of final diagnosis; ^b^ p-value of numeric variables by Kruskal Wallis; ^c^ p-value of categorized variables by chi2; percentage by row (column ‘Number total of cases’; empty cell is because any participant had this characteristic; ^d^High grade (grade I-II) and Low grade (grade III-IV).
